# Acceptance and commitment therapy versus mindfulness-based stress reduction for newly diagnosed head and neck cancer patients: A randomized controlled trial assessing efficacy for positive psychology, depression, anxiety, and quality of life

**DOI:** 10.1371/journal.pone.0267887

**Published:** 2022-05-10

**Authors:** Zheng Zhang, Mohammad Farris Iman Leong Bin Abdullah, Nurul Izzah Shari, Ping Lu

**Affiliations:** 1 Department of Community Health, Advanced Medical and Dental Institute, Universiti Sains Malaysia, Kepala Batas, Pulau Pinang, Malaysia; 2 School of Human Resource Development and Psychology, Faculty of Social Sciences and Humanities, Universiti Teknologi Malaysia, Skudai, Johor, Malaysia; 3 Department of Oncology, 1^st^ Affiliated Hospital, Xinxiang Medical University, Henan, People’s Republic of China; University of Hong Kong, HONG KONG

## Abstract

**Background and aim:**

Head and neck cancer patients are vulnerable to various psychological complications due to the effects of both cancer itself and cancer treatment on patients’ appearance and physical well-being. Nevertheless, few data have been obtained on effective psychosocial interventions that could protect this group of cancer patients’ psychological well-being. Therefore, this three-armed, parallel-group, double-blind, randomized control trial (RCT) aims to evaluate and compare the effects of acceptance and commitment therapy (ACT) and mindfulness-based stress reduction (MBSR) on positive psychology (such as posttraumatic growth [PTG], hope, and optimism), quality of life (QoL), and psychological complications (depression, anxiety, and experiential avoidance) among newly diagnosed head and neck cancer patients.

**Methods and analysis:**

This RCT will target newly diagnosed head and neck cancer patients who have been treated only with surgery or who have not yet received any treatment. In total, 120 patients who meet all of the study’s inclusion criteria and none of its exclusion criteria will be randomly assigned into three groups—an ACT group, an MBSR group, and a treatment-as-usual control group—at a 1:1:1 allocation ratio. Participants in the two intervention groups (the ACT and MBSR groups) will undergo an eight-week group intervention program. During this program, each intervention will comprise eight modules based on ACT and MBSR, respectively. Outcome assessments will be performed across a three-point timeline, including before the intervention (t_0_), immediately after the psychosocial intervention at eight weeks (t_1_), and six months after the intervention (t_2_). The primary outcome that will be assessed during this RCT is PTG. Meanwhile, the secondary outcomes that will be evaluated in this study are such as QoL, hope, optimism, depression, anxiety, and experiential avoidance.

**Trial registration number:**

NCT04800419 (ClinicalTrials.gov). Registered on March 16, 2021.

## Introduction

### Head and neck cancer and its associated psychological issues

Head and neck cancer is a group of biologically similar cancers that affect the oral cavity, lips, nasal cavity, paranasal sinuses, pharynx, and larynx. It affects 550,000 people worldwide annually, leading to 300,000 deaths each year [1]. In Malaysia, 9,419 head and neck cancer cases were reported between 2007 and 2011, accounting for 9.1% of all cancer diagnoses [2]. Therefore, head and neck cancer is the country’s fourth-most-prevalent cancer [2]. Head and neck cancer leads to high incidence of various psychosocial issues among cancer survivors, such as depression, suicide, interpersonal relationship conflict, facial disfigurement, damage to self-image, social isolation, and continued substance dependence and abuse [3]. These psychosocial issues may lead to negative consequences in head and neck cancer survivors. For example facial disfigurement can increase depression and reduce quality of life (QoL) among head and neck cancer patients [4].

Some studies have found that head and neck cancer is associated with the highest documented rates of depression and anxiety compared with other cancer types [5]. Depression and anxiety could reduce head and neck cancer patients’ QoL [6]. A 2003 article by Spiegel and Giese-Davis pointed out that depression may predict cancer’s progression and mortality through a bidirectional relationship between depression and cancer; depression and cancer can negatively affect each other [7]. Consequently, depression and anxiety among head and neck cancer patients should be adequately treated.

*Experiential avoidance* (EA) has been broadly defined as attempts to avoid thoughts, feelings, memories, physical sensations, and other internal experiences—even when such avoidance causes long-term harm [8]. Currently, negative thoughts, emotions, and sensations are believed not to contribute to psychiatric illnesses; however, a habitual and persistent unwillingness to experience uncomfortable thoughts and feelings (and the associated avoidance and inhibition of these experiences) is thought to be linked to psychiatric illnesses [8, 9]. Additionally, reduced EA also indirectly predicts a higher QoL among cancer patients via its effect on depression symptoms [10]. Therefore, an intervention that successfully reduces EA is predicted to also reduce depression and anxiety symptoms.

#### Posttraumatic growth, hope, and optimism’s influence on quality of life

Despite the negative psychological complications associated with head and neck cancer and its treatment, psychology research has shifted focus in recent years, examining the positive aspects and growth resulting from traumatic experiences of cancer and cancer patients’ QoL. Among the positive psychological experiences which may develop in cancer patients include posttraumatic growth, hope, and optimism. Posttraumatic growth (PTG) describes positive psychological changes experienced as the result of a struggle due to a life-threatening crisis or event. PTG comprises five components, and an individual who has experienced more PTG will better appreciate life and have better interpersonal relationships, more personal strength, higher levels of spiritual development, and/or experience more possibilities in life compared with before development of PTG [11]. PTG results in positive psychological changes beyond pre-trauma levels [11]. It has been shown to be present among head and neck cancer patients [12, 13]. PTG is important and should be emphasized as a positive psychological characteristic among cancer patients because it inversely correlates with depression and psychological distress [14, 15]. It also positively correlates with health-related QoL [15, 16]. However, it should be noted that not all patients with trauma related to living with cancer could trigger the development of PTG. If the traumatic experience of living with cancer is too intense, it may lead to failure for cognitive reprocessing of the traumatic event to search for meaning, which will hamper the development of PTG [17]. Hence, it is pivotal to investigate psychosocial intervention which effectively facilitate the development of PTG.

*Hope* is a positive, goal-directed motivational state and a dispositional trait that enables a tendency to adopt a positive outlook on life [18]. It comprises two components: *agency*, which is the perceived motivation to pursue and achieve goals, and *pathway*, which is the perceived ability to generate approaches to achieving goals [18]. Hope is associated with several positive outcomes for cancer patients. For instance, hope is negatively associated with depression, anxiety, and psychological distress among cancer patients [19–22]. Higher levels of hope are associated with better QoL and spiritual well-being for cancer patients, and it is the most significant factor associated with greater posttraumatic growth among cancer patients compared with other positive psychology elements [23–25].

Similarly, *optimism* is the stable and consistent belief that good things—rather than bad things—will happen in life [26]. Optimism has been shown to positively correlate with psychological well-being and inversely correlate with depression and psychological distress [27]. It is also positively associated with health-related QoL among cancer patients [27–29]. In of head and neck cancer patients’ context, optimism predicts better one-year survival rates independently from confounding demographic and clinical factors [30]. QoL is an important measure and health indicator in the field of psycho-oncology for assessments and treatment outcomes [31]. Since head and neck cancer is associated with a wide range of illness complications and adverse treatment effects that can reduce QoL [32–34], psychosocial interventions that could enhance head and neck cancer patients’ QoL must be investigated.

#### Acceptance and commitment therapy and mindfulness-based stress reduction

We have identified two psychosocial interventions that could enhance cancer’s positive psychological sequelae and reduce its negative psychological sequelae: acceptance and commitment therapy (ACT) and mindfulness-based stress reduction (MBSR). ACT is a third-generation cognitive behavioral approach that uses acceptance and mindfulness processes, as well as commitment and behavioral change processes, to generate psychological flexibility [9]. Psychological flexibility is the ability to act on the long-term chosen values in life and accept undesired thoughts, feelings and short-term impulses stemming from distressing life events. Hence, psychological flexibility allows one to control EA, which may be utilized to cope with distressing life events [35].

ACT is useful for cancer patients because it can help them handle the negative emotions resulting from cancer (such as uncertainty, anxiety, sadness, and anger), rather than avoiding these emotions [36, 37]. A meta-analysis of 25 treatment-control comparison studies with a total sample size of 2,256 cancer patients found that ACT greatly affected psychological distress, particularly reducing depression and anxiety symptoms among cancer patients [38]. ACT also greatly increased QoL and hope among cancer patients [38]. Although that study’s sample size may be too small to fully justify ACT’s significant effect on hope, ACT’s main principles that help cancer patients clarify their values and commit to actions are believed to improve their hope [38]. Moreover, an RCT of 410 colorectal cancer survivors reported that ACT significantly increased PTG at six-month and 12-month periods of a telephone-based health coaching intervention using ACT strategies [39]. However, to date, ACT’s effect on PTG, hope, optimism, acceptance, depression, anxiety, and QoL among head and neck cancer patients has not been investigated.

MBSR is an eight-week, standardized group intervention comprising mindfulness meditation and gentle yoga designed to address stress, pain, and illness [40]. Cancer patients’ participation in MBSR has been shown to have increase in spirituality, PTG, self-compassion, and positive states of mind [41–47]. Studies have emphasized that MBSR increases PTG among participating cancer patients immediately after an eight-week intervention [44, 47, 48]. A meta-analysis of 29 studies with a total of 3,476 cancer survivors evaluated mindfulness-based interventions’ efficacy in facilitating mental well-being, revealing that MBSR significantly reduced depression, anxiety, stress, and fatigue while enhancing PTG, QoL, and mindfulness among cancer patients [49]. Moreover, MBSR has been reported to lower psychological distress and improve total, social, and emotional QoL among head and neck cancer patients [50]. To date, however, MBSR’s effect on cancer patients’ hope has not been investigated. Therefore, an investigation of MBSR’s effects on PTG, hope, optimism, depression, anxiety, and QoL among head and neck cancer patients would be interesting. Moreover, a comparison of MBSR’s efficacy compared to ACT would be valuable.

#### Rationale

Head and neck cancer differs from other cancer types due to various psychosocial issues associated with it [3, 4]. Moreover, the devastating complications of both cancer itself and cancer treatments’ side effects—such as fatigue, pain, speech and swallowing problems, breathing problems, mucositis, xerostomia, and trismus—further disrupt many functions and daily living activities, increasing psychological distress and decreasing QoL [32, 33]. Therefore, an investigation of the psychosocial interventions that could enhance PTG, hope, optimism, and QoL among head and neck cancer patients is crucial. Reducing these patients’ internalized stigma and EA could also ultimately restore these cancer survivors’ mental and physical well-being. ACT and MBSR have been reported to enhance positive psychology and alleviate psychological distress among cancer patients [38, 49]. However, data regarding ACT and MBSR’s effects on positive psychology, psychological sequelae and QoL in head and neck cancer patients are lacking. ACT’s effects on PTG, hope, optimism, QoL, depression, anxiety, and EA among head and neck cancer patients have not been studied. Although MBSR has been reported to lower psychological distress and enhance QoL in head and neck cancer patients, its effects on positive psychology and EA have not been investigated. Hence, MBSR may act as a good comparator intervention to ACT for evaluating its efficacy on positive psychology, psychological sequelae and QoL among head and neck cancer patients. Therefore, we have proposed a three-armed RCT to evaluate these effects over time compared to a control group receiving treatment-as-usual.

### Objectives

#### Primary objectives

1. Evaluate ACT’s efficacy in enhancing PTG among head and neck cancer patients compared to MBSR and a control (treatment-as-usual) immediately after the eight-week intervention and 24 weeks post-intervention compared to pre-intervention

#### Secondary objectives

1. Examine ACT’s efficacy in enhancing positive psychology (such as hope and optimism) among head and neck cancer patients compared to MBSR and a control (treatment-as-usual) immediately after the eight-week intervention and 24 weeks post-intervention compared to pre-intervention
2. Examine ACT’s efficacy in reducing EA among head and neck cancer patients compared to MBSR and a control (no treatment-as-usual) immediately after the eight-week intervention and 24 weeks post-intervention compared to pre-intervention
3. Examine ACT’s efficacy in reducing psychological complications (depression and anxiety) among head and neck cancer patients compared to MBSR and a control (treatment-as-usual) immediately after the eight-week intervention and 24 weeks after intervention compared to pre-intervention.
4. Evaluate ACT’s efficacy in enhancing QoL among head and neck cancer patients compared to MBSR and a control (treatment-as-usual) immediately after the eight-week intervention and 24 weeks post-intervention compared to pre-intervention.

## Methods/Design

### Design

The current study will conduct a multicentre, three-armed, parallel-group, double-blind RCT that is expected to run for three years.

### Setting

This study will be conducted in Advanced Medical and Dental Institute (AMDI) at the Universiti Sains Malaysia (USM) and Universiti Kebangsaan Malaysia Medical Centre (UKMMC). AMDI, USM is a tertiary referral center for oncology in Peninsular Malaysia while UKMMC is a tertiary referral center for various illnesses, including oncology cases in Peninsular Malaysia.

### Support and ethics

This study has received approval from the Human Research Ethics Committee of Universiti Sains Malaysia (code: USM/JEPeM/21040321). The study will be conducted in compliance with the *Declaration of Helsinki* (1974) and its amendments, as well as the *Malaysian Good Clinical Practice Guidelines for Clinical Trials*. The institutional review board will be informed of any modification to the study’s protocol (such as its eligibility criteria, study procedures, outcome measures, scheduled interventions, and data analyses) in writing. This manuscript was written without any deviation from the original protocol submitted to the Human Research Ethics Committee of Universiti Sains Malaysia except we added more details to the title in this manuscript to make it more informative. The title of the original manuscript reads “Randomized controlled trial of acceptance and commitment therapy versus mindfulness-based stress reduction in newly diagnosed head and neck cancer patients”, while the title of this manuscript reads “Acceptance and commitment therapy versus mindfulness-based stress reduction for newly diagnosed head and neck cancer patients: A randomized controlled trial assessing efficacy for positive psychology, depression, anxiety, and quality of life”.

The principal investigator will lead the trial center and closely coordinate with the research coordinator and the site coordinator, participate in subject recruitment, and oversee the study’s consent procedures. A trial monitoring committee has been established. This committee is chaired by the principal investigator, and it will meet weekly to manage the study on a day-to-day basis, audit trial conduct, and prepare reports for submission to the Human Research Ethics Committee of Universiti Sains Malaysia. The trial’s data monitoring and auditing will be conducted by the AMDI’s clinical trial coordination unit at the Universiti Sains Malaysia—independently from the trial’s funder.

All participants will be permitted to withdraw from the study at any time without specifying the reason for their withdrawal, and we will not use any of the information collected from participants who choose to withdraw. Prior to their participation in this study, participants will be informed that the results of this study will only be used for research purposes and will not be recorded in patients’ case files. Participants’ personally identifiable information will not be sought or recorded, and participants’ anonymity will be assured. Each participant will be sequentially assigned a research number (e.g., *RCT001*, *RCT002*, etc.). All documents involved in this study’s assessment of research subjects—including participants’ personal information (socio-demographic, substance history, symptomatology, and questionnaire responses)—will be stored in document files and locked in a cabinet whose key is kept by the corresponding author.

Participants may also withdraw from the study if an adverse event occurs. An *adverse event* (AE) is any untoward medical occurrence involving a participant in a trial intervention that does not necessarily causally relate to this treatment. An AE can be any unfavorable or unintended sign, symptom, or disease that is temporarily associated with the use of an investigational intervention, whether related or unrelated to the investigational intervention. Participants will be issued a study card featuring the research team’s contact details and encouraged to maintain close contact by phone and report any AE. If an AE occurs, the event will be reported in the “Adverse Event” section of the study’s case report form (CRF), and a serious adverse event report will be filled out if necessary (S1 Fig). Such reports would include the AE’s name, date of onset and date of recovery, severity, relationship to the studied intervention, measures undertaken regarding the studied intervention, treatment, and outcome (whether resolved or ongoing). All serious AEs will be reported to the Human Research Ethics Committee of Universiti Sains Malaysia. AE-related criteria that would exclude participants from the study are:

1. The presence of adverse reactions unrelated to the study that cause subjects to feel uncomfortable continuing this study
2. The presence of adverse reactions that may be related to the study, such as an unusual illness that started after a studied intervention
3. Unusual changes in participants’ behavior, temperament, routine, suicidal tendency, or psychotic symptoms that began after a studied intervention
4. Any suspected or unexpected adverse events inconsistent with the generally accepted administration of the ACT and MBSR; in general, psychotherapy patients should not exhibit any side effects or health-detrimental effects

If an unexpectedly serious AE is reported in a subject or more in any of the randomized intervention group (ACT or MBSR), an interim analysis will be conducted to investigate this matter. If the findings of this investigation indicate any safety concerns which is related to the intervention administered (ACT or MBSR), the Trial Monitoring Committee will terminate the trial prematurely. Serious AE which are indication for premature termination of trial include suicidal tendency, life-threatening self-harm, and hospitalization related to psychological adverse events arise from the intervention administered.

Only the study’s corresponding author and co-authors are permitted to access the study’s files for data analysis or publication purposes. The findings of this study will be submitted for publication in peer-reviewed academic journals and for presentation at international conferences and symposia. The study’s principal investigator will be listed as the corresponding author, and authorship eligibility will be based on the International Committee of Medical Journal Editors’ recommendations.

All of this study’s investigators declare no financial or other competing interests in conducting this study.

### Participants

This study’s participants will be recruited from the source population. This population includes all newly diagnosed head and neck cancer patients who have been treated only with surgery or who have not yet received treatment and who are registered under the oncology and otorhinolaryngology unit of the Advanced Medical and Dental Institute (AMDI) at the Universiti Sains Malaysia (USM) or the oncology and otorhinolaryngology unit of Universiti Kebangsaan Malaysia Medical Centre (UKMMC). The study’s sample size was determined based on G-Power 3.1.9.2 for analysis of variance (ANOVA). This sample size was calculated based on continuous responses to the posttraumatic growth inventory used in the study by Labelle et al. (2015) [48] with a medium effect size (0.15) and a two-tailed alpha of 0.05. The results of this calculation indicated that the current study requires a total sample of 93 for three equal-sized groups to achieve a power of 0.8. Anticipating a drop-out rate of 30%, we estimated a total sample size of 120 respondents and 40 respondents for each group.

### Inclusion criteria

The study’s inclusion criteria will include: (1) patients who have been newly diagnosed with head and neck cancer (within the previous month), whose diagnosis has been confirmed by a histopathological examination report, who have been treated only with surgery or have yet to received treatment, and who are at any cancer stage except metastasis to the central nervous system; (2) patients aged 18 years and older; (3) patients who have undergone surgery and plan to receive the standard regime of radiotherapy or chemotherapy; (4) patients who have developed depression and anxiety symptoms after their head and neck cancer diagnosis with a “Hospital Anxiety and Depression Scale” (HADS) depression subscale score of ≥ 8 and an anxiety subscale score of ≥ 8; and (5) patients who are able to read and understand written Malay.

### Exclusion criteria

Patients will be excluded if they: (1) have a history of pre-existing psychiatric illness; (2) have a history of illicit drug use, substance use disorder, alcohol use disorder, substance-related disorder, or alcohol-related disorder (patients will be screened via urine dipstick for drugs and the “Mini International Neuropsychiatric Interview” to exclude these conditions); (3) have a history of medical illness that can induce psychiatric symptoms; (4) currently receive any psychotherapy or counseling; (5) are physically unfit for this study’s interventions (for example those who are bed-bound or too weak to engage in the study interventions); (6) exhibit cognitive impairment (patients will be screened via the “Mini Mental State Examination” and patients who receive a score of < 24/30 will be excluded); (7) have received any pharmacotherapy for anxiety and depression symptoms developed after their cancer diagnosis; and (8) are pregnant.

### Recruitment process

This study will use a consecutive sampling method. The research team will approach head and neck cancer patients registered at the two targeted institutions and explain the study’s objectives and procedures. Patients who are interested in participating in this study will be screened for inclusion and exclusion criteria by a research team member. All eligible patients will be invited to participate in this study. The study’s purposes and procedures will be thoroughly explained (verbal explanation by the research assistant and a copy of the participant information will also be distributed) to prospective participants before they are invited to participate in this study, their anonymity will be assured, and they will be informed of their right to withdraw from the study at any time and the data collected will be discarded. Eligible patients will be given 48 hours to decide on their participation in the study. Then, participants will sign written informed consent to participate in the study before they enroll in the study.

### Randomization

This study will use stratified permuted block randomization, stratifying trial participants according to their age (*18 to 40 years old*, *41 to 60 years old*, and *over 60 years old*) and gender (*male* and *female*). Participants will be randomized into three groups: an ACT group, an MBSR group, and a control group of patients on treatment-as-usual for intervention. Using a 1:1:1 allocation ratio and block randomization, we will randomly assign participants to the three groups. An allocation sequence will be generated via computer-generated random numbers, which will be obtained by a research assistant who is not otherwise involved in this study. The allocation sequence will be concealed in an opaque, sequentially numbered envelope.

### Interventions

An eight-week psychosocial intervention will be administered to participants in each intervention group (MBSR and ACT) while participants in the control group will receive treatment-as-usual. The study’s outcomes will be assessed at three times: pre-intervention (t_0_), immediately after the eight-week intervention (t_1_), and 24 weeks after the intervention (t_2_).

Both the ACT and MBSR interventions will comprise eight sessions delivered at a rate of one session per week. Each session will last for one hour. At AMDI, USM, and UKMMC, chemotherapy for head and neck cancer lasts for 8 weeks, delivered at a rate of one session per week. Similarly, radiotherapy for head and neck cancer lasts for eight weeks at these institutions. Therefore, this study’s ACT and MBSR sessions will align with participants’ chemotherapy or radiotherapy sessions to ensure that all participants attend their intervention sessions. ACT and MBSR will be provided in group settings with intervention groups of 10 participants each.

#### Acceptance and commitment therapy (ACT)

ACT utilize two main approaches to facilitate the development of psychological flexibility, such as mindfulness and acceptance as well as commitment and behavioral change. ACT session begin with building of therapeutic rapport and formulation of the case. Brief mindfulness exercise and home assignment review are also introduced at the initial stage. The remaining sessions of ACT will then focus on the use of pre-plan learning materials and exercises to facilitate didactic and experiential learning. Hence, each subsequent sessions will work on addressing the specific themes introduced by Hayes et al. (2009), such as EA, cognitive fusion, self as context, defining valued directions in life, and the commitment to action strategies to pursue their valued directions in life [9]. Home assignments in the form of behavioral practices and experiments are provided at the end of each session. The workbook provided to the respondent is based on the book “Get out of your mind and into your life” written by Hayes et al (2005) [51] and “ACT made simple” written by Harris (2009) [52]. The details of all the ACT sessions are described in S1 Appendix.

#### Mindfulness-based stress reduction (MBSR)

MBSR will be conducted as group sessions, with each session lasts for 2.5 hours, once a week for 8 weeks with another 45 minutes of home practice on daily basis. The MBSR sessions will be based on therapy format developed by Kabat-Zinn [40]. All the sessions focus on mindfulness practices, sharing of experiences with others, and didactic teaching on stress. Mindfulness practices which will be discussed include body scan exercise, and mindful breathing meditation techniques, three-minute breathing exercises, five-minute seeing or hearing exercises, bodily mindfulness in movement and mindful stretching, yoga and sitting meditation. The therapists will review feedbacks for each session, acknowledge the thoughts, feelings, and senses, examine how participants practice mindfulness, and give assignments to encourage participants to continually practice mindfulness at home. Each participant will receive a CD detailing on meditation practices and a workbook on the mindfulness practices. The details of all the MBSR sessions are described in S1 Appendix.

#### Treatment-as-usual control group

The participants in the control group will receive treatment-as-usual in which non-specific ingredients of the psychotherapeutic approach will be administered, such as psychological understanding to the management of an individual patient, identifying current problems, providing opportunities for disclosure, reassurance, and deep breathing exercise. They will be given equal amount of time and attention from the professional figure compared to the intervention groups, whereby they will also attach to an 8-session program (with one session per week for 8 weeks).

### Treatment fidelity

ACT will be conducted for the ACT intervention group by a therapist trained in ACT, who will have a trained backup therapist on standby to replace the primary therapist when necessary. Similarly, MBSR will be conducted for the MBSR group by a therapist trained in MBSR, and a trained backup therapist will be on standby to replace the primary therapist when necessary. All the study’s therapists and backup therapists will be postgraduate students in psychology who are not otherwise involved in the study. All the therapists have two-year experience of conducting psychotherapy. They will also receive training manual detailing the psychotherapy sessions for both ACT and MBSR, and they will also undergo 2 full days of brief training workshop for ACT and MBSR from the principal investigator. As for monitoring of treatment integrity, a psychiatrist and a clinical psychologist, both trained in ACT and MBSR will measure the delivery of the interventions by all the therapists in the ACT and MBSR groups independently. 15% of audio-recording of the psychotherapy sessions will be randomly selected and stratified according to therapist and the phase of intervention (early, middle or end) and then, treatment integrity assessments will be performed by using: (1) the Drexel University CT/ACT Therapist Adherence and Competence Rating Scale (DUACRS) [53] for ACT sessions and (2) the Mindfulness-Based Interventions: Teaching Assessment Criteria (MBI:TAC) [54]. Each treatment integrity assessor will assess half of the selected sessions. Finally, the interrater reliability of the two treatment integrity assessors is computed to assess the treatment integrity of all the therapists in delivering the interventions [55]. In addition, the principal investigator will discuss issues regarding the delivery of the interventions with the therapist who is inconsistent in conducting the ACT and MBSR sessions.

### Blinding

Participants will be kept unaware of the study’s randomization into the designated groups, conducted by a research assistant who is not otherwise involved in the study and concealed in an opaque, sequentially numbered envelope. Therefore, participants will not know which group they are allocated to. In addition, the intervention groups (ACT and MBSR) as well as the treatment-as-usual control group will receive an 8-session program (with one session per week for 8 weeks) and participants in the treatment-as-usual group will also receive equal amount of attention and time from the professional figure who administer the program.

The researchers will also be “blinded” for the study since the participants’ randomized assignment into the designated groups will be conducted by a research assistant who is not otherwise involved in the study or data analysis. This project’s data collection will also be conducted by that research assistant who is not otherwise involved in the study or data analysis and who is unaware of the study’s hypotheses. Moreover, the project’s data analysis will be conducted by statisticians who are not otherwise involved in the study. A statistical analysis plan is established prior to the final unblinded of the data lock.

### Measures

**Fig 1:**
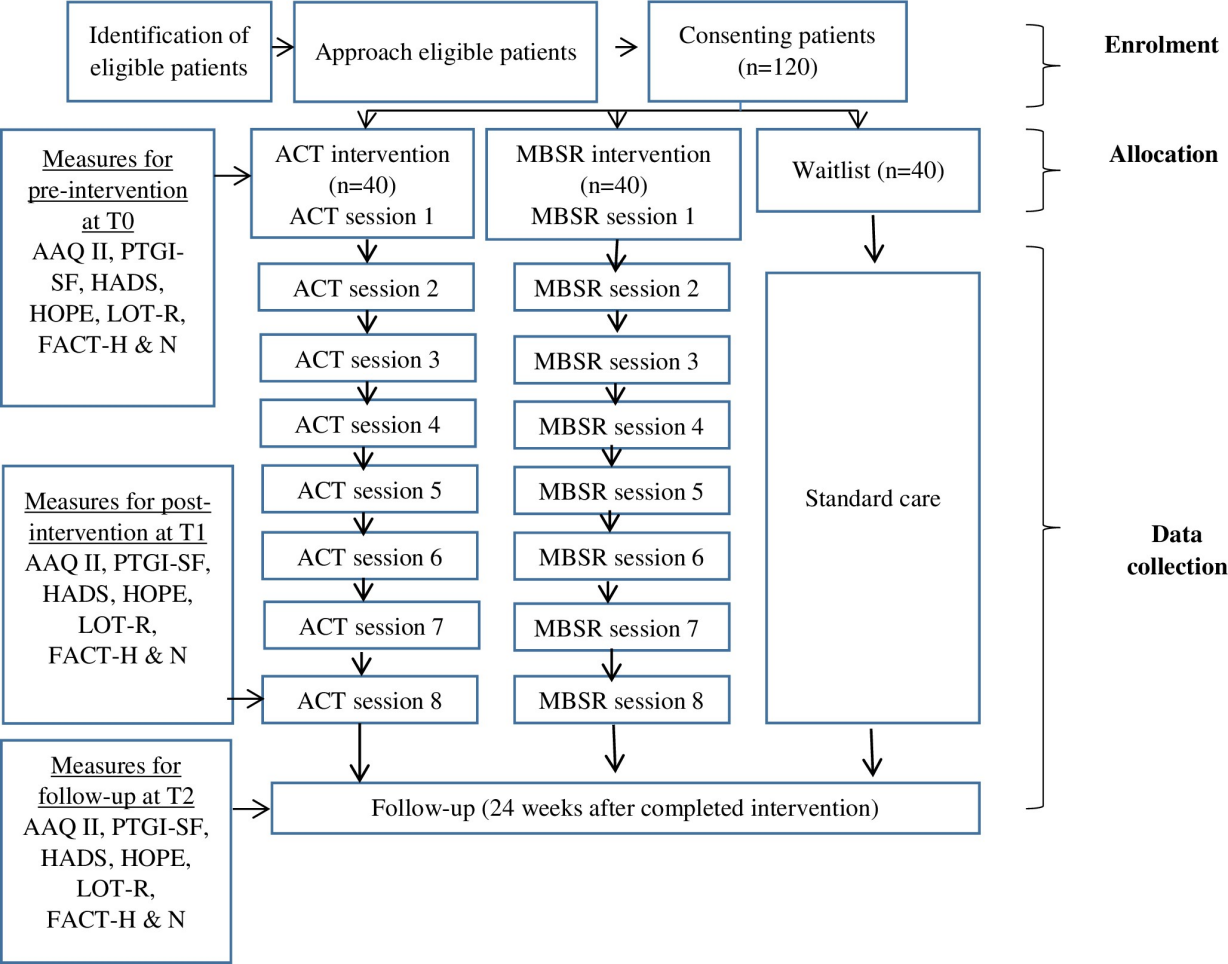
The flow diagram of the study procedures and outcome measures. ACT = acceptance and commitment therapy, MBSR = mindfulness-based stress reduction, AAQ-II = Acceptance and Action Questionnaire version II, PTGI-SF = Posttraumatic Growth Inventory-Short Form, HADS = Hospital Anxiety and Depression Scale, HOPE = Hope Scale, LOT-R = Life Orientation Test-Revised, FACT-H & N = Functional Assessment of Cancer Therapy–Head & Neck, T_0_ = pre-intervention assessment, T_1_ = immediately post-intervention at 8 weeks, T_2_ = 24 weeks after intervention.

Fig 1 is a flowchart summarizing this study’s procedures and outcome measures. The scheduled assessment of the study’s outcome measures is illustrated in Table 1. Data collection will be conducted every weekday during working hours, and flyers announcing the study and the benefits of participating in this study will be disseminated to all newly diagnosed head and neck cancer patients at the study’s focal institutions (within one month of their diagnosis) in order to ensure adequate subject enrolment to achieve the calculated sample size. This clinical trial protocol was written according to the “Standard Protocol Items: Recommendations for Intervention Trials 2013.”

**Table 1:** Scheduled assessment of the randomized control trial’s outcome measures.

	Enrolment	Allocation	Post-allocation	
Time	*-t*_*1*_	*t*_*0*_ *(0 week)*	*t*_*1*_ *(8 weeks)*	*t*_*2*_ *(24 weeks)*
**Enrolment:**				
**Eligibility screen**	X			
**Informed consent**	X			
**Allocation**		X	X	X
**Interventions:**				
**(1) Acceptance and commitment therapy (ACT)**		X	X	X
**(2) Mindfulness-based stress reduction (MBSR)**		X	X	X
**(3) Controls (treatment-as-usual)**		X	X	X
**Assessments:**				
**(1) Socio-demographic and tumor characteristics data**		X		
**(2) Primary outcomes:**				
**“Posttraumatic Growth Inventory–Short Form” (PTGI-SF)**		X	X	X
**(3) Secondary outcomes:**				
**(a) “Dispositional Hope Scale”**		X	X	X
**(b) “Life Orientation Test–Revised” (LOT-R)**		X	X	X
**(c) “Acceptance and Action Questionnaire” (AAQ-II)**		X	X	X
**(d) “Hospital Anxiety and Depression Scale” (HADS)**		X	X	X
**(e) “Functional Assessment of Cancer Therapy–Head & Neck” (FACT-H & N)**		X	X	X

#### Sociodemographic and tumor characteristics

The sociodemographic characteristics which will be recorded from each participant include age, gender, ethnicity, marital status, education, and employment status. While the tumor characteristics which will be gathered from the participants are duration of cancer diagnosis, types of head and neck cancer, stage of cancer and modalities of cancer treatment received.

#### Primary outcome

*Posttraumatic growth*. The Malay version of the “Posttraumatic Growth Inventory–Short Form” (PTGI-SF) will be used to measure degrees of posttraumatic growth among participants. The PTGI-SF is a shorter version of the original PTGI, and it comprises 10 items, two of which measure each of the five PTG factors. The higher an individual’s PTGI-SF score, the more posttraumatic growth they have achieved. The PTGI can be substituted by the PTGI-SF with little loss of information [56]. The Malay version of the PTGI-SF was validated among the Malaysian cancer patient population, and it has good internal consistency with a Cronbach’s alpha of 0.887 and a Cronbach’s alpha of the five PTG factors ranging from 0.700 to 0.813 [57].

#### Secondary outcomes

*Quality of life*. The Malay version of “Functional Assessment of Cancer Therapy–Head & Neck” (FACT–H & N) will be used to assess quality of life among participants. FACT-H & N is a self-reported tool comprising 39 items and five subscales: *physical well-being* (seven items), *social/family well-being* (seven items), *emotional well-being* (six items), *functional well-being* (seven items), and *head & neck cancer additional concerns* (12 items). Each item is scored on a five-point Likert scale ranging from 0 (*not at all*) to 4 (*very much*). The higher an individual’s FACT–H & N score, the greater their QoL. The tool has also registered excellent psychometric properties [58]. FACT–H & N has been translated and validated among the Malaysian cancer patient population. All of its subscales have moderate to good internal consistency with Cronbach’s alphas ranging from 0.65 to 0.87 [59].

*Hope*. The Malay version of the “Dispositional Hope Scale” will be used to measure participants’ degrees of hope. The “Dispositional Hope Scale” is a self-rated, 12-item scale that assesses respondents’ hope levels. It comprises two domains: *agency* and *pathway*. Four of the scale’s 12 items assess agency, while another four items assess pathways. The four other items are fillers. Each item is scored using a Likert scale ranging from *definitely false* to *definitely true* [60]. The Malay version of the Scale was validated among the Malaysian cancer patient population, and it has good internal consistency with a Cronbach’s alpha of 0.716 [61].

*Optimism*. The Malay version of the “Life Orientation Test–Revised (LOT-R)” will be used to evaluate degrees of optimism among participants. The LOT-R is a self-rated tool comprising six items in two domains, *optimism* and *pessimism*. Each domain is made up of three items. The tool has good internal consistency and has been found to be stable over time [26]. The Malay version of the LOT-R was validated among the Malaysian cancer patient population, and it has acceptable internal consistency [62].

*Experiential avoidance*. The Malay version of the “Acceptance and Action Questionnaire” (AAQ-II) will be used to measure degrees of experiential avoidance or psychological inflexibility among participants. The AAQ-II is the questionnaire’s second version, revised from the original version, and it is shorter (seven items) while offering better psychometric consistency. Participants’ AAQ-II scores will be calculated by summing up the questionnaire’s seven items. Higher scores indicate higher levels of psychological inflexibility [63]. Validation of the Malay version of the AAQ-II has indicated that the tool has excellent internal consistency with a Cronbach’s alpha of 0.91, and it has been found to be a unidimensional scale that measures psychological inflexibility/EA [64].

*Depression and anxiety*. The Malay version of the “Hospital Anxiety and Depression Scale” (HADS) will be used to assess the severity of depression and anxiety symptoms among participants. The HADS is a 14-item, self-rated questionnaire with seven items in each of its subscales. Each item is scored from 0 to 3, and the total score for both the depression and anxiety subscales ranges from 0 to 21 per subscale [65]. The cut-off for depression cases is 8/21, and the cut-off for anxiety cases is also 8/21 [66]. The Malay version of the HADS has been validated among Malaysian breast cancer patients, and it exhibited acceptable to good internal consistency for its total score and subscales with Cronbach’s alphas ranging from 0.73 to 0.87 [67].

#### Other measures

The participants compliance to the interventions will be recorded as they will be requested to fill in electronic dairies regarding their performance of the home assignments and practice of the interventions at home. The reasons for absence from therapy sessions, dropouts and loss to follow up (such as loss of interest to participate, dislike of the intervention, not feeling well or death) will also be recorded.

### Data analysis

All of this study’s data analysis will be conducted using the Statistical Package for Social Sciences, version 26 (SPSS 26; SPSS Inc., Chicago, Illinois, USA). Initially, the baseline sociodemographic and tumor characteristics will be presented by randomized groups (ACT, MBSR, and control groups).

The mean difference in the primary outcome (total PTGI-SF score) for the three randomized groups (ACT, MBSR, and control groups) at each specific time point (pre-intervention [t_0_], post-treatment at 8 weeks [t_1_] and 24 weeks after intervention [t_2_]) will be assessed using one way analysis of variance (ANOVA) followed by false discovery rate adjustment. For the main pool analysis, mixed linear model will be employed for comparing the primary outcome (total PTGI-SF score) between ACT and control groups and between MBSR and control groups across the three time points (t_0_, t_1_ and t_2_), while controlling for stratification factors such as age and gender. The main effects of intervention in the groups and time points will be presented as estimated marginal mean and standard error of mean. The study’s primary analysis will follow the intention-to-treat (ITT) principle. The data analysis for the study’s secondary outcomes (quality of life, hope, optimism, depression, anxiety, and EA) will be conducted similarly to the primary outcomes’ calculation. In addition, 95% confidence interval will also be presented alongside the main effect. Statistical significance will be two-tailed and set to *p* < 0.05.

To handle any missing data, if the missing data represent less than 5% of the study’s total collected data, they will be ignored. If the missing data represent more than 5% but less than 40% of the total collected and are assumed to be randomly missing, then multiple imputation (restricted maximum likelihood estimation) will be performed using Stata 15. However, if the missing data represent more than 40% of the total collected data or are assumed to be missing either not randomly or completely randomly, then only the collected data will be used for the study’s analysis, and the missing data will be explained as a research limitation in any publications of the study’s findings [68].

## Discussion

This study will primarily investigate the efficacy of two psychosocial interventions (ACT and MBSR) in enhancing positive psychology elements (such as PTG). Secondarily, it will evaluate these interventions’ efficacy in reducing psychological complications (such as depression, anxiety, and EA), enhancing other positive psychology elements (such as hope and optimism), and increasing vital outcome (such as quality of life) among newly diagnosed head and neck cancer patients. To the best of our knowledge, this study will be the first to investigate and compare ACT’s and MBSR’s effects in improving psychological well-being among head and neck cancer patients.

This study emphasize on investigating how ACT and MBSR affect changes in posttraumatic growth as the primary outcome as posttraumatic growth represents a unique transitional process characterized by several positive psychological outcomes (such as better appreciation of life, positive relationship with others, higher spiritual development, greater personal strength, and enhanced new possibilities in life) beyond the level of attainment prior to the traumatic event of living with cancer and contribute to psychological and physical well-being of cancer survivors [69]. In essence, posttraumatic growth is associated with enhancement of quality of life among cancer survivors.

This study will provide comprehensive scientific evidence to guide treating clinicians’ efficacious psychosocial interventions in order to enhance positive psychology elements and diminish psychological complications, safeguarding head and neck cancer patients’ mental well-being. If MBSR and ACT effectively facilitate mental well-being among head and neck cancer patients, then these psychosocial interventions should be included in the treatment regime for this patient group. This study will also contribute to future research since its methodology can be replicated to investigate ACT’s and MBSR’s effects on psychological complications and other positive psychology elements among patients with other cancer types.

Since this study will only utilize a maximum of four therapists to conduct the intervention sessions, this may limit the generalizability of the study findings. Hence, it is of utmost importance to train up more therapists for future randomized clinical trials which investigate efficacy of ACT and to deliver this intervention to cancer patients in oncology settings. The first step in training for therapists is to set up ACT workshop which will allow ACT trainee to experience the metaphors and exercises used in ACT on a personal context and as a receiver of ACT. This step of organizing ACT workshop for trainee therapists serve several benefits and advantages. Participants in ACT workshop exhibited not only increase in knowledge regarding ACT, but also develop interest for further learning and utilize ACT concepts and utilize ACT practices in their clinical work [70]. Moreover, trainee therapists should be encouraged to join their supervisors in regular individual or group ACT sessions in addition to their regular supervision hours. During supervision hours, trainee therapists are encouraged to conceptualize a case based on the ACT perspective and report on the progress of the case on a weekly basis. The core learning objectives for the trainee therapists should be based on three principles: (1) to develop a sense of personal wholeness and able to reflect the same sense to the patients who experience EA, (2) to be able to help patients to accept emotions and thoughts by reflecting their own experience to the experience of the patients, and (3) to be able to guide patients to understand the costs of EA on their personal values in life, so that patients are willing to commit substantially towards actions to pursue their values in life.

In addition, since this study only recruit Malaysia head and neck cancer patients, future RCT with larger sample size (under medium to large effect size) and patients recruited from multiple countries is warranted to evaluate the effects of ACT and MBSR on positive psychology (such as PTG, hope optimism), quality of life and negative outcomes (such as depression, anxiety and EA).

## Supporting information

S1 Checklist Standard Protocol Items: Recommendations for Intervention Trials 2013 (SPIRIT) checklist. (DOC)

S1 Fig Flow chart of adverse event reporting. (TIF)

S1 Appendix The detail descriptions of the ACT and MBSR sessions. (DOCX)

S2 Appendix Participant information sheet and consent form (Malay and English versions). (DOC)

S3 Appendix Research protocol approved by the human research ethics committee. (DOCX)
