# Supplementary figures and images for: Acceptance and commitment therapy versus mindfulness-based stress reduction for newly diagnosed head and neck cancer patients: A randomized controlled trial assessing efficacy for positive psychology, depression, anxiety, and quality of life
Source: PLoS One. 2022 May 10;17(5):e0267887. doi: 10.1371/journal.pone.0267887 (PMC9089868; doi:10.1371/journal.pone.0267887)

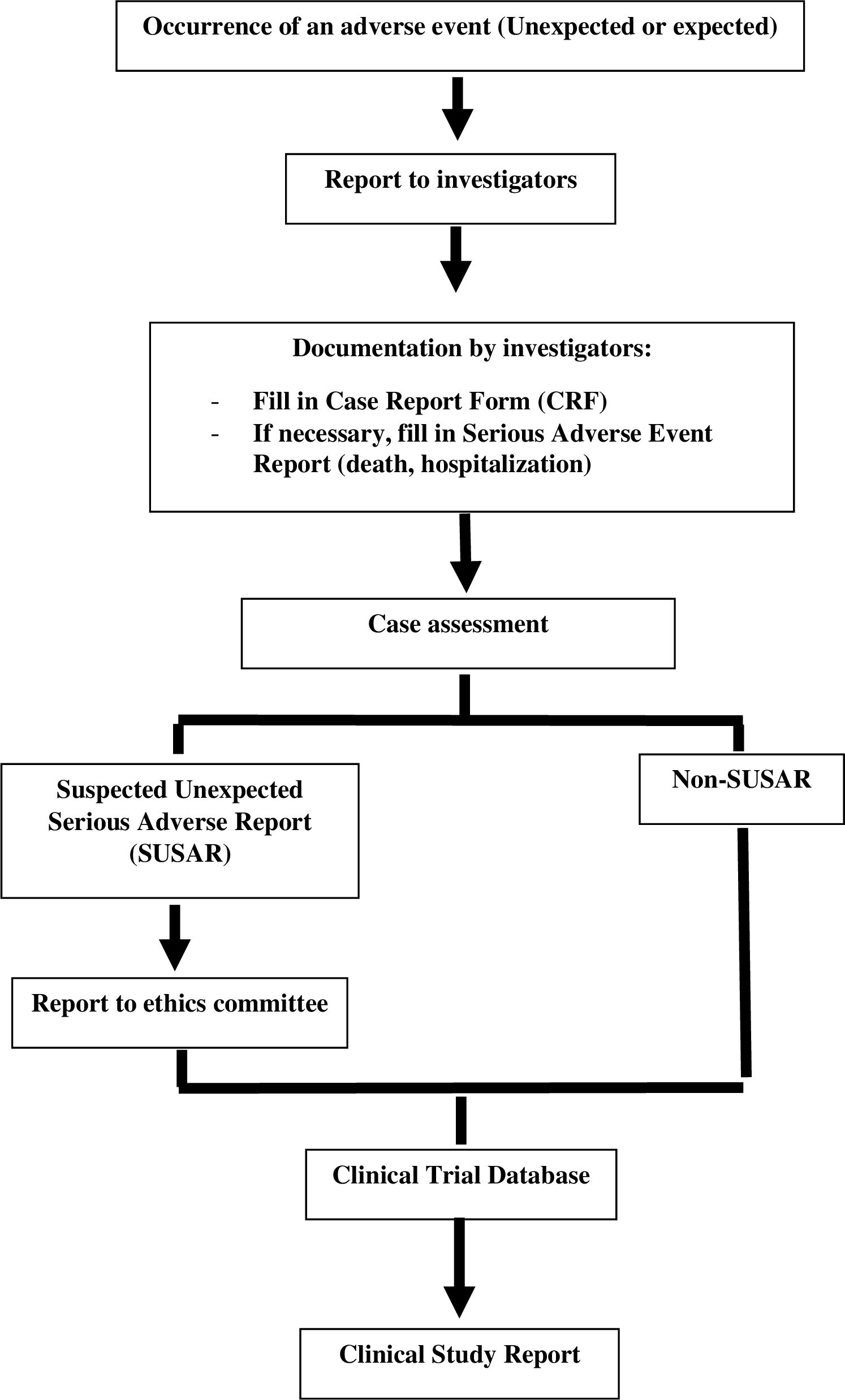

Supplement: S1 Fig — (TIF) [file pone.0267887.s002.tif]
