# Supplementary material for: Acceptance and commitment therapy versus mindfulness-based stress reduction for newly diagnosed head and neck cancer patients: A randomized controlled trial assessing efficacy for positive psychology, depression, anxiety, and quality of life
Source: PLoS One. 2022 May 10;17(5):e0267887. doi: 10.1371/journal.pone.0267887 (PMC9089868; doi:10.1371/journal.pone.0267887)
Supplement: S1 Appendix — (DOCX) [file pone.0267887.s003.docx]

**S1 Appendix: The detail descriptions of the ACT and MBSR sessions**

**Acceptance and commitment therapy (ACT)**

*Session 1*

The first ACT session will start with the therapist attempt to develop therapeutic relationship with the respondents and an initial intake interview with the respondents. Respondents’ personal information—such as their main external barriers, past fusions, EA, disruptions, strengths, resources, and past life history—will be obtained. Based on the information provided, the study’s ACT therapist will generate case formulations. Then, these case formulations will be used to map respondents’ life issues on matrix diagrams.

*Session 2*

The study’s ACT therapist will start the second ACT session by discussing issues that arose during the previous session. Then, respondents will be encouraged to embrace their unpleasant feelings and thoughts (creative hopelessness). The therapist will suggest new possibilities—such as the idea that unpleasant feelings and thoughts are not patients’ enemies but, rather, that control is patients’ main problem. They will suggest that the alternative to control is the willingness to “give space” and “sit” with unpleasant thoughts, feelings, and memories. Then, respondents will be encouraged to actively “contact” their psychological experiences without struggling, to “sit with” and make space for their experiences, and to remain in the present moment. Several metaphors will be used in this encouragement, such as a *tug of war with a monster* and physicalizing unpleasant thoughts and feelings. Such metaphors will help respondents recognize their EA and “let go” of unhelpful emotion-control strategies.

*Session 3*

During the third ACT session, the therapist will discuss on defusion with the respondents. Defusion will help reduce behavioral avoidance resulting from unpleasant emotions and thoughts. This defusion will aim to increase the extent to which respondents’ can choose actions based on their values, rather than being controlled by their emotions and thoughts. The therapist will use metaphors such as the *hands trap*, *the mind as a bully*, and *passengers on a bus*, and they will perform a paper-pushing exercise with respondents. As homework, the therapist will encourage respondents to practice physicalizing exercises and use the *passengers on a bus* metaphor to address unwanted thoughts and feelings.

*Session 4*

During the third ACT session, the ACT therapist will guide respondents in practicing structured mindfulness exercises, particularly mindful breathing. Through these mindfulness exercises, the therapist will encourage respondents to focus on the present moment. For example, while breathing, respondents will be encouraged to use their senses by focusing on inhaling and exhaling, noticing the air temperature, and feeling their muscles move while they breathe in and out. Then, their awareness will be expanded through the therapist’s instructions to notice the external environment, such as sights and sounds, sensations of bodily contact, and posture. Next, respondents will be asked to breathe mindfully while simultaneously acknowledging the presence of their unpleasant thoughts and feelings. The therapist will encourage respondents to experience these thoughts and feeling with openness, interest, and receptiveness—without attempting to change these thoughts and feelings. Instead of trying to distract respondents from their unpleasant feelings and thoughts, this activity aims to awaken respondents to the many occurrences that they can appreciate and enjoy, rather than focusing excessively on their unpleasant feelings and thoughts. As respondents become more aware, they can respond efficiently—particularly when experiencing unpleasant feelings and thoughts. The therapist will use the metaphors of *an emotional storm* and *dropping an anchor*. With these metaphors, the therapist will emphasize that dropping an anchor does not make a storm stop but does hold a boat firm; the storm will come and go in its own time. For this week’s homework, respondents will be encouraged to practice mindfulness using mindful breathing at least twice daily—after waking in the morning and before going to sleep. Respondents will also be required to record their mindfulness exercises in a mindfulness worksheet.

*Session 5*

During the fifth ACT session, the ACT therapist will review respondents’ mindfulness exercise worksheets and ask about their experiences practicing mindfulness. Then, the therapist will encourage respondents to use mindfulness in their daily activities, such as mindful eating and mindful bathing.

*Session 6*

The sixth ACT session will aim to develop respondents’ sense of self as a context. Respondents will learn that the self is safe, continuous, consistent, and separate from their unpleasant thoughts and feelings. Moreover, respondents will be asked to develop a position from which they can observe and then release their unpleasant thoughts and feelings. Again, the ACT therapist will use metaphors to guide respondents. This sessions metaphor’s will include *a chessboard* and *a furnished room* to ensure that respondents understand the concept of the self as a context.

*Session 7*

The seventh ACT session will involve therapeutic processes and particularly Hexaflex values. This session will aim to ensure that respondents have clearly expressed their values. The ACT therapist will help respondents clarify their values and identify their life priorities, such as relationships, health, education, and spirituality. *Values* are understood as the anticipated qualities of ongoing actions that are freely chosen and do not require evaluation. *Values* differ from *goals*; once values are identified, goals can be established. Some exercises—such as value assessments, a value compass, and a bullseye—will be conducted to help respondents differentiate between their values and their goals, as well as identify and evaluate whether their life engages with their values or not.

*Session 8*

During the eighth and final ACT session, the ACT therapist will again discuss respondents’ values determined during Session 7. Session 8 will aim to ensure that respondents can link their values to their action strategies. The therapist will ask respondents to list feasible actions and strategies. Finally, the therapist will encourage respondents to take at least minimal steps or actions that could pursue their life values.

**Mindfulness-based stress reduction (MBSR)**

*Session 1*

In the first MBSR session, the study’s MBSR therapist will explain mindfulness-based therapy and the importance of home practice to respondents. Home practice time is a challenging part of this course, but the outcomes are worthwhile. Then, the therapist will conduct an automatic pilot activity. This activity aims to increase awareness, encouraging respondents to respond to their situations by choice, rather than reacting automatically. This pilot will show that people tend to be only dimly aware of what they are doing, following unhelpful thinking habits that lead to stress. The therapist will conduct a “raisin exercise” to show how people’s attention is not always fully directed at the present moment. Then, the therapist will emphasize that when people become more aware of the present moment, including their thoughts, feelings, and bodily sensations, they can enjoy greater freedom and choice. The therapist will explain that mindfulness does not aim for a specific result but, rather, increased awareness of where one is and how one allows themselves to be. At the end of this session, the therapist will introduce respondents to the body-scan exercise. In this exercise, respondents will be encouraged to focus on the different parts of their bodies and use each part of their body to anchor their awareness of a moment. Before the session ends, the therapist will ask respondents to practice this body-scan exercise six times at home while listening to a take-home CD which explain how the exercise is performed.

*Session 2*

During the second MBSR session, the therapist will review the previous session’s lessons and home practices. The therapist will discuss the body scan exercise, meditation techniques, and mindful breathing meditation techniques with respondents. Finally, the therapist will give respondents assignments on the techniques learned above.

*Session 3*

In the third MBSR session, the therapist will review the assignments and lessons of Session 2, practice sitting meditations, and collect feedback from respondents regarding this session. The therapist will practice three-minute breathing exercises with respondents and give respondents home assignments regarding what was learned in this session.

*Session 4*

During the fourth MBSR session, the therapist will review respondents’ home practices, conduct five-minute seeing or hearing exercises, and teach bodily mindfulness in movement and mindful stretching. Then, the therapist will explain that the almost inevitable bodily discomfort that arises when practicing mindful stretching provides an ideal opportunity to learn how to approach difficult and unwanted feelings with curiosity, gentleness, kindness, and courage. The therapist will practice the breathing space with respondents. Finally, the therapist will assign a home practice of mindful movement using a CD track daily for six days.

*Session 5*

The therapist will start the fifth MBSR session by reviewing the assignments and lessons from Session 4. Then, they will help respondents practice standing yoga and sitting meditation. Next, the therapist will discuss how to respond instead of reacting. The core MBSR skill is learning to replace unconscious stress reactions with conscious stress responses. For example, when respondents practice sitting meditation regularly, they can notice when their awareness drifts and how to gently refocus their attention. Thus, respondents can become more aware and respond mindfully, rather than reacting automatically.

*Session 6*

During the sixth MBSR session, the therapist will discuss the previous session’s lesson and encourage respondents to use their bodies to increase their awareness. By remaining aware of the present moment, they can mindfully respond instead of automatically reacting to stressors. However, such responses require that respondents experience and accept stressors in the present moment. Then, respondents will walk mindfully and perform a three-minute breathing space exercise.

*Session 7*

In the seventh MBSR session, the therapist will discuss the lessons of Session 6 and assess patients’ sleep, QoL, and mental health—including their unwanted thoughts and feelings. Then, the therapist will explain that thoughts are not facts but mental events. The therapist will ask respondents to use breathing space to cope with unwanted thoughts.

*Session 8*

During the eighth and final MBSR session, the therapist will summarize the previous sessions’ content and encourage respondents to continue regular mindfulness exercises. After reconnecting with expanded awareness, the therapist will also encourage respondents to undertake a considered action to deal with troubling feelings, such as performing a pleasurable activity or an activity that will instill a sense of satisfaction.
