# Supplementary material for: Acceptance and commitment therapy versus mindfulness-based stress reduction for newly diagnosed head and neck cancer patients: A randomized controlled trial assessing efficacy for positive psychology, depression, anxiety, and quality of life
Source: PLoS One. 2022 May 10;17(5):e0267887. doi: 10.1371/journal.pone.0267887 (PMC9089868; doi:10.1371/journal.pone.0267887)
Supplement: S2 Appendix — (DOC) [file pone.0267887.s004.doc]

**LAMPIRAN A**

**MAKLUMAT KAJIAN**

**Tajuk Kajian : Kajian Rawak Terkawal Perbandingan Terapi Penerimaan dan Komitmen Dengan Pengurangan Tekanan Berdasarkan Kesedaran Dalam Kalangan Pesakit Kanser Kepala dan Leher yang Baru Didiagnosis**

Nama Penyelidik dan penyelidik bersama : Dr. Zhang Zheng, Dr. Mohammad Farris Iman Leong Bin Abdullah (MMC: 43103), Dr. Nurul Izzah Shari, Profesor Dr. Lu Ping

# PENGENALAN

Anda adalah dipelawa untuk menyertai satu kajian intervensi secara sukarela. Kajian ini adalah berkaitan dengan penilaian keberkesanan dua jenis psikoterapi, iaitu terapi penerimaan dan komitmen (ACT) dan pengurangan tekanan berdasarkan kesedaran (MBSR) untuk meningkatkan aspek psikologi positif seperti perkembangan pasca trauma, optimis, harapan dan kualiti kehidupan serta mengurangkan stigma, pengelakan pengalaman buruk, kemurungan dan keresahan dalam kalangan pesakit kanser kepala dan leher yang baru didiagnosis.

Adalah penting bagi anda membaca dan memahami maklumat kajian sebelum anda bersetuju untuk menyertai kajian penyelidikan ini. Sekiranya anda menyertai kajian ini, anda akan menerima satu salinan borang ini untuk simpanan anda.

Penyertaan anda di dalam kajian ini dijangka mengambil masa 8 bulan. Seramai 120 orang dijangka akan menyertai kajian ini.

#### TUJUAN KAJIAN

Kajian ini bertujuan untuk membandingkan keberkesanan terapi penerimaan dan komitmen (ACT), pengurangan tekanan berdasarkan kesedaran (MBSR) and subjek kawalan untuk meningkatkan aspek psikologi positif seperti perkembangan pasca trauma, optimis, harapan dan kualiti kehidupan serta mengurangkan stigma, pengelakan pengalaman buruk, kemurungan dan keresahan dalam kalangan pesakit kanser kepala dan leher yang baru didiagnosis merentasi 3 titik masa, iaitu sebelum intevensi, sejurus selepas tamatnya intervensi dan 6 bulan lepas tamatnya intervensi.

KELAYAKAN PENYERTAAN

Salah seorang kakitangan kajian akan membincangkan kelayakan untuk menyertai kajian ini. Adalah penting anda berterus terang kakitangan tersebut [Jika berkaitan boleh ditambah “termasuk sejarah kesihatan anda].

Kajian ini akan melibatkan individu yang:

(1) Baru didiagnosis dengan kanser kepala dan leher yang telah dirawat dengan surgeri atau belum dirawat, kecuali mereka yang mempunyai metastasis ke bahagian otak.

(2) mana-mana tahap

(3) 18 tahun dan ke atas

(4) Boleh faham dan tulis dalam Bahasa Melayu dan Bahasa Inggeris

(5) Merancang untuk menjalani rawatan kemoterapi

(6) Mereka yang mempunyai gejala kemurungan dan keresahan selepas diagnosis kanser kepala dan leher dengan skor HADS-subskala kemurungan ≥ 8 dan subskala keresahan ≥ 8

Kajian ini tidak akan melibatkan individu yang:

(1) mempunyai sejarah penyakit mental

(2) mempunyai sejarah penggunaan dadah dan kebergantungan alkohol

(3) mempunyai sejarah penyakit medikal yang boleh memyebabkan simptom

(4) tengah dirawat dengan psikoterapi atau sesi kaunseling

(5) keadaan fizikal yang lemah dan tidak boleh melakukan intervensi

(6) mempuyai kemerosotan kognisi dengan skor MMSE < 24/30.

#### PROSEDUR-PROSEDUR KAJIAN

Anda akan menjawab soal selidik yang akan diberi kepada anda oleh penolong penyelidik kami pada tiga titik masa yang berbeza, iaitu T0 (kali pertama), T1 (8 minggu kemudian) dan T2 (24 minggu kemudian). Soal selidik yang akan dijawab adalah seperti berikut:

(i) Soal selidik sosiodemografi dan klinikal yang merangkumi butiran tentang umur, jantina, status perkahwinan, pekerjaan, tahap pendidikan, pendapatan bulanan, agama, tahap kanser dan jenis kanser (pada T0 sahaja),

(ii) Inventori Perkembangan Pasca Trauma-Borang Pendek (PTGI-SF) yang menilai tahap perkembangan pasca trauma.

(iii) Skala Harapan yang menilai tahap harapan

(iv) Ujian Orientasi Kehidupan-Pindaan (LOT-R) yang menilai tahap optimis

(v) Soal Selidik Penerimaan dan Tindakan (AAQ-II) yang menilai tahap pengelakan pengalaman buruk

(vi) Penilaian Fungsi Terapi Kanser- Kepala dan Leher (FACT-H & N) yang menilai tahap kualiti kehidupan

(vii) Skala Keresahan dan Kemurungan Hospital (HADS) yang menilai tahap simptom kemurungan dan keresahan

Para peserta akan diaturkan ke tiga kumpulan intervensi oleh sistem komputer kami secara rawak. Intervensi yang akan diberi adalah sama ada terapi penerimaan dan komitmen (ACT) atau pengurangan tekanan berdasarkan kesedaran (MBSR). Tempoh intervensi adalah selama 8 minggu.

(1) Terapi penerimaan dan komitmen (ACT): ia merupakan kaedah kelakuan kognisi generasi ketiga yang meggunakan proses penerimaan dan kesedaran, komitmen dan perubahan tingkahlaku untuk membentuk fleksibiliti psikologi. Tidak seperti CBT yang sasarkan perubahan fikiran negative dan emosi, ACT direka untuk meningkatkan adaptasi pada masalah kehidupan melalui penerimaan, defuse kognisi, kesedaran dan senaman membentuk perspektif untuk menyokong pesakit kanser menguruskan kelakuan mereka dengan nilai individu mereka. Justeru, ACT merangkumi 8 sesi, dimana satu sesi dalam setiap minggu untuk membantu pesakit membentuk kelakuan kesihatan yang lebih kondusif dimana halangan dalaman seperti emosi yang mengganggu dan fikiran yang mengurangkan keberkesanan diri akan disasarkan.

(2) Pengurangan tekanan berdasarkan kesedaran (MBSR): Ia adalah program berasaskan program selama lapan minggu yang menawarkan latihan kesedaran intensif sekular untuk membantu individu yang mengalami tekanan, kegelisahan, kemurungan dan kesakitan. Ia menggunakan kombinasi meditasi kesedaran, kesedaran tubuh, yoga dan penerokaan pola tingkah laku, pemikiran, perasaan dan tindakan. Istilah “kesedaran” digunakan untuk merujuk pada keadaan kesedaran psikologi, kelakuan yang mempromosikan kesedaran ini, iaitu cara memproses maklumat dan sifat watak. Program MBSR adalah program lapan minggu yang melibatkan latihan dalam meditasi minda dan yoga. Individu biasanya bertemu dengan ahli terapi sekali seminggu. Kursus MBSR standard terdiri daripada lapan, sesi mingguan sebanyak 2 setengah jam hingga 3 jam setiap satu, ditambah dengan sesi sepanjang hari selepas minggu keenam yang merangkumi satu hari latihan penuh. Setiap sesi dimulakan dengan latihan kesedaran yang telah dipelajari oleh individu semasa sesi mingguan. Latihan utama program MBSR adalah imbasan badan, yoga lembut dan meditasi duduk. Melalui program ini, para peserta akan melakukan salah satu latihan ini setiap hari. Di samping itu, terdapat pelbagai latihan berpusat yang memasukkan amalan kesedaran dalam kehidupan seharian. Latihan lain pula boleh melatih kesedaran dengan penuh perhatian berkaitan dengan pemikiran, sensasi dan emosi. Setiap sesi memfokuskan pada satu topik tertentu yang diterokai dengan latihan khusus, serta pembentangan daripada pengajar dan dialog antara peserta kumpulan. Terdapat juga masa bagi para peserta untuk berkongsi apa yang mereka alami semasa latihan dan juga untuk membincangkan berkaitan amalan kesedaran harian mereka di luar kelas.

##### RISIKO

Penyertaan dalam kajian ini akan membawa kepada risiko yang minima. Namun, jika anda mengalami gangguan emosi selepas menjawab soal selidik, kami akan mencadangkan rujukan kepada kaunselor di Hospital Bukit Mertajam, Pulau Pinang. Jika anda masih mengalami tanda-tanda kemurungan dan keresahan selepas tamatnya penyertaan dalam kajian ini, kami akan mencadangkan rujukan ke Klinik Minda Sihat, Institut Perubatan dan Pergigian Termaju, Universiti Sains Malaysia, Pulau Pinang untuk mendapat penilaian dan rawatan yang sewajarnya. Sila maklumkan kepada kakitangan kajian sekiranya anda menghadapi sebarang masalah atau mempunyai sebarang maklumat penting yang mungkin mengubah persetujuan anda untuk terus menyertai kajian ini.

MELAPORKAN PENGALAMAN KESIHATAN (Jika Kajian Melibatkan Kesihatan SAHAJA)

Sila hubungi kakitangan berikut pada bila-bila masa sekiranya anda mengalami sebarang masalah kesihatan, samada berkaitan atau tidak berkaitan dengan kajian ini.

Dr. <Mohammad Farris Iman Leong Bin Abdullah> [No. Pendaftaran Penuh Majlis Perubatan Malaysia: 43103 ] di talian <04-5622482> atau <018-6669950> secepat mungkin.

PENYERTAAN DALAM KAJIAN

Penyertaan anda dalam kajian ini adalah secara sukarela. Anda berhak menolak untuk menyertai kajian ini atau menamatkan penyertaan anda pada bila-bila masa, tanpa sebarang kehilangan manfaat yang sepatutnya anda perolehi.

Penyertaan anda juga mungkin boleh diberhentikan oleh kakitangan kajian ini tanpa persetujuan anda sekiranya anda didapati tidak sesuai untuk meneruskan kajian ini berdasarkan protokol kajian. Kakitangan kajian akan memaklumkan anda sekiranya anda perlu diberhentikan dari menyertai kajian ini.

MANFAAT YANG MUNGKIN [Manfaat terhadap Individu, Masyarakat, Universiti]

Prosedur kajian ini akan diberikan kepada anda tanpa kos. Anda boleh menerima maklumat tentang status kesihatan mental anda dan juga tahap ciri-ciri psikologi positif anda yang dijangka penting untuk mengekalkan kesihatan mental anda selepas menghidap kanser dan mengalami komplikasi yang berkaitan dengannya. Anda juga dapat mengikuti sesi psikoterapi terapi penerimaan dan komitmen (ACT) atau pengurangan tekanan berdasarkan kesedaran (MBSR) yang dijangka mungkin dapat membantu dalam mengekalkan kesihatan mental anda dalam menghadapi penyakit kanser.

Hasil kajian ini diharapkan, dapat memberi manfaat kepada masyarakat umum untuk membuktikan bahawa kedua-dua intervensi psikoterapi ini dapat meningkatkan aspek psikologi positif dan kualiti kehidupan serta mengawal komplikasi psikologi kanser seperti stigma terhadap kanser, pengelakan pengalaman buruk, kemurungan dan keresahan. Ini akan mengesyorkan integrasi kedua-dua intervensi ini dalam regim rawatan untuk pesakit kanser kepala dan leher dimana data berkenaan dengan keberkesanan kedua-dua intervensi ini dalam kalangan pesakit kanser kepala dan leher adalah agak berkurangan.

Anda tidak akan menerima sebarang pampasan kerana menyertai kajian ini. Namun sebarang keperluan perjalanan berkaitan dengan penyertaan ini akan diberikan.

PERSOALAN

Sekiranya anda mempunyai sebarang soalan mengenai prosedur kajian ini atau hak-hak anda, sila hubungi;

Dr. Zhang Zheng

Penyelidik Utama

Jabatan Kesihatan Komuniti

Institut Perubatan dan Pergigian Termaju

Universiti Sains Malaysia

SAINS@BERTAM

13200 Kepala Batas

Pulau Pinang

04-5622482

Dr. Mohammad Farris Iman Leong Bin Abdullah

Penyelidik Utama

Jabatan Kesihatan Komuniti

Institut Perubatan dan Pergigian Termaju

Universiti Sains Malaysia

SAINS@BERTAM

13200 Kepala Batas

Pulau Pinang

018-6669950

Sekiranya anda mempunyai sebarang soalan berkaitan kelulusan Etika atau sebarang pertanyaan dan masalah berkaitan kajian ini, sila hubungi;

En. Mohd Bazlan Hafidz Mukrim

Setiausaha Jawatankuasa Etika Penyelidikan (Manusia) USM

Bahagian Penyelidikan dan Inovasi (P&I)

USM Kampus Kesihatan.

No. Tel: 09-767 2354 / 09-767 2362

Email : [bazlan@usm.my](mailto:bazlan@usm.my)

ATAU

Cik Nor Amira Khurshid Ahmed

Sekretariat Jawatankuasa Etika Penyelidikan (Manusia) USM

Pejabat Pengurusan dan Kreativiti Penyelidikan (RCMO)

USM Kampus Induk, Pulau Pinang.

No. Tel: 04-6536537

Email: noramira@usm.my

KERAHSIAAN

Maklumat yang anda berikan akan dirahsiakan oleh kakitangan kajian. Ianya tidak akan dedahkan secara umum melainkan jika ia dikehendaki oleh undang-undang.

Data yang diperolehi dari kajian ini tidak akan mengenalpasti anda secara perseorangan. Hasil kajian mungkin akan diterbitkan untuk tujuan perkongsian ilmu.

Semua borang kajian dan data yang anda berikan termasuk rekod perubatan anda yang asal mungkin akan disemak oleh pihak penyelidik, Lembaga Etika kajian ini dan pihak berkuasa regulatori bagi tujuan mengesahkan prosedur dan/atau data kajian klinikal. Maklumat anda akan disimpan dalam komputer dan hanya kakitangan kajian yang dibolehkan sahaja dibenarkan untuk mendapatkan dan memproses data tersebut. Maklumat anda akan disimpan selama tujuh tahun selepas tamatnya kajian ini sebelum dipadam dan dimusnahkan sepenuhnya.

Dengan menandatangani borang persetujuan ini, anda membenarkan penelitian rekod, penyimpanan maklumat dan pemprosesan data seperti yang dihuraikan di atas.

TANDATANGAN

Untuk dimasukkan ke dalam kajian ini, anda atau wakil sah anda mesti menandatangani serta mencatatkan tarikh halaman tandatangan (Lihat contoh Borang Keizinan Peserta di LAMPIRAN S atau LAMPIRAN P).

LAMPIRAN S

Borang Keizinan Peserta

(Halaman Tandatangan)

***Tajuk Kajian:* Kajian Rawak Terkawal Perbandingan Terapi Penerimaan dan Komitmen Dengan Pengurangan Tekanan Berdasarkan Kesedaran Dalam Kalangan Pesakit Kanser Kepala dan Leher yang Baru Didiagnosis**

***Nama Penyelidik: Dr. Zhang Zheng, Dr. Mohammad Farris Iman Leong Bin Abdullah (MMC: 43103), Dr. Nurul Izzah Shari, Profesor Dr. Lu Ping***

Untuk menyertai kajian ini, anda atau wakil sah anda mesti menandatangani mukasurat ini. Dengan menandatangani mukasurat ini, saya mengesahkan yang berikut:

- Saya telah membaca semua maklumat dalam Borang Maklumat dan Keizinan Pesakit ini termasuk apa-apa maklumat berkaitan risiko yang ada dalam kajian dan saya telah pun diberi masa yang mencukupi untuk mempertimbangkan maklumat tersebut.
- Semua soalan-soalan saya telah dijawab dengan memuaskan.
- Saya, secara sukarela, bersetuju menyertai kajian penyelidikan ini, mematuhi segala prosedur kajian dan memberi maklumat yang diperlukan kepada doktor, para jururawat dan juga kakitangan lain yang berkaitan apabila diminta.
- Saya boleh menamatkan penyertaan saya dalam kajian ini pada bila-bila masa.
- Saya telah pun menerima satu salinan Borang Maklumat dan Keizinan Peserta untuk simpanan peribadi saya.

Nama Peserta

No. Kad Pengenalan Peserta

**Tandatangan Peserta** atau Wakil Sah **Tarikh** (dd/MM/yy)

(Masa jika perlu)

Nama & Tandatangan Individu yang Mengendalikan Tarikh (dd/MM/yy)

Perbincangan Keizinan

Nama Saksi dan Tandatangan Tarikh (dd/MM/yy)

Nota: i) Semua peserta yang mengambil bahagian dalam projek penyelidikan ini tidak dilindungi insuran.

LAMPIRAN P

Borang Keizinan bagi Penerbitan Bahan yang berkaitan dengan Peserta Kajian

(Halaman Tandatangan)

***Tajuk Kajian:* Kajian Rawak Terkawal Perbandingan Terapi Penerimaan dan Komitmen Dengan Pengurangan Tekanan Berdasarkan Kesedaran Dalam Kalangan Pesakit Kanser Kepala dan Leher yang Baru Didiagnosis**

***Nama Penyelidik: Dr. Zhang Zheng, Dr. Mohammad Farris Iman Leong Bin Abdullah (MMC: 43103), Dr. Nurul Izzah Shari, Profesor Dr. Lu Ping.***

Untuk menyertai kajian ini, anda atau wakil sah anda mesti menandatangani mukasurat ini.

Dengan menandatangani mukasurat ini, saya memahami yang berikut:

- Bahan yang akan diterbitkan tanpa dilampirkan dengan nama saya dan setiap percubaan yang akan dibuat untuk memastikan ketanpanamaan saya. Saya memahami, walaubagaimanapun, ketanpanamaan yang sempurna tidak dapat dijamin. Kemungkinan sesiapa yang menjaga saya di hospital atau saudara dapat mengenali saya.
- Bahan yang akan diterbitkan dalam penerbitan mingguan/bulanan/dwibulanan/suku tahunan/dwi tahunan merupakan satu penyebaran yang luas dan tersebar ke seluruh dunia. Kebanyakan penerbitan ini akan tersebar kepada doktor-doktor dan juga bukan doktor termasuk ahli sains dan ahli jurnal.
- Bahan tersebut juga akan dilampirkan pada laman web jurnal di seluruh dunia. Sesetengah laman web ini bebas dikunjungi oleh semua orang.
- Bahan tersebut juga akan digunakan sebagai penerbitan tempatan dan disampaikan oleh ramai doktor dan ahli sains di seluruh dunia.
- Bahan tersebut juga akan digunakan sebagai penerbitan buku oleh penerbit jurnal.
- Bahan tersebut tidak akan digunakan untuk pengiklanan ataupun bahan untuk membungkus.

Saya juga memberi keizinan bahawa bahan tersebut boleh digunakan sebagai penerbitan lain yang diminta oleh penerbit dengan kriteria berikut:

- Bahan tersebut tidak akan digunakan untuk pengiklanan atau bahan untuk membungkus.
- Bahan tersebut tidak akan digunakan di luar konteks – contohnya: Gambar tidak akan digunakan untuk menggambarkan sesuatu artikel yang tidak berkaitan dengan subjek dalam foto tersebut.

Nama Peserta

No. Kad Pengenalan Peserta T/tangan Peserta Tarikh (dd/MM/yy)

Nama & Tandatangan Individu yang Mengendalikan Tarikh (dd/MM/yy)

Perbincangan Keizinan

Nota: i) Semua peserta yang mengambil bahagian dalam projek penyelidikan ini tidak dilindungi insuran.

CONTOH ATTACHMENT B

RESEARCH INFORMATION

*Research Title:* Randomized controlled trial of acceptance and commitment therapy versus mindfulness-based stress reduction in newly diagnosed head and neck cancer patients

*Name of main and co-Researcher: Dr. Zhang Zheng, Dr. Mohammad Farris Iman Leong Bin Abdullah (MMC: 43103), Dr. Nurul Izzah Shari, Profesor Dr. Lu Ping*

#### INTRODUCTION

You are invited to take part voluntarily in an interventional research. This research is about evaluating the efficacy of two types of psychotherapy, that are acceptance and commitment therapy (ACT) and mindfulness-based stress reduction (MBSR) to enhance the positive psychology, such as posttraumatic growth, optimism, hope, and quality of life as well as alleviating the internalized stigma, experiential avoidance, depression and anxiety among newly diagnosed head and neck cancer patients.

It is important that you read and understand this research information before agreeing to participate in this study. You will receive a copy of this form to keep for your records if you agree to participate.

Your participation in this study is expected to last for 8 months. This study is estimated to include up to 120 participants.

#### PURPOSE OF THE STUDY

The purpose of this study are to compare the efficacy of acceptance and commitment therapy (ACT), mindfulness-based stress reduction (MBSR), and control group to enhance enhance the positive psychology, such as posttraumatic growth, optimism, hope, and quality of life as well as alleviating the internalized stigma, experiential avoidance, depression and anxiety among newly diagnosed head and neck cancer patients across three time-frame, which are before intervention, immediately after completion of the intervention and 6 months after completion of the intervention.

#### PARTICIPANTS CRITERIA

The research team members will discussed your eligibility to participate in this study. It is important that you are completely truthful with the staff including your health history.

This study will include individual who are:

(1) newly diagnosed with head and neck cancer who have been treated only with surgery or still remain untreated, except for those with metastasis to the central nervous system.

(2) any stage of cancer

(3) age 18 years and above.

(4) Can understand and write in English or Malay.

(5) planning for the standard regime of chemotherapy.

(6) having depression and anxiety symptoms after diagnosis with head and neck cancer with Hospital Anxiety and Depression Scale (HADS)- Depression subscale score of ≥ 8 and HADS- Anxiety subscale score of ≥ 8.

This study will not incude individual who are:

(1) having history of pre-existing psychiatric illnesses

(2) having history of illicit drug intake and alcohol dependence

(3) hacing history of medical illnesses which can induced psychiatric symptoms

(4) currently on any psychotherapy or counselling sessions.

(5) are physically unfit to perform intervention.

(6) exhibiting cognitive impairment (patients are screen with Mini Mental State Examination, in which those with score of < 24/30 will be excluded).

STUDY PROCEDURES

You will be administered questionnaires by our research assistant at three different time-frame, which is T0 (first time), T1 (8 weeks later), and T2 (24 weeks later). The questionnaires which will be administered to you include:

(i) Socio-demographic and clinical questionnaire which includes age, gender, marital status, employment, education, monthly income, religion, stage of cancer and type of head and neck cancer (only during T0).

(ii) The Posttraumatic Growth Inventory-Short Form (PTGI-SF) to assess the degree of PTG of the participants

(iii) The Dispositional Hope Scale to assess the degree of hope of the participants

(iv) The Life Orientation Test-Revised (LOT-R) to assess the degree of optimism

(v) The Acceptance and Action Questionnaire (AAQ-II) to assess the degree of experiential avoidance

(vi) The Functional Assessment of Cancer Therapy – Head & Neck (FACT-H & N) to assess the degree of quality of life

(vii) The Hospital Anxiety and Depression Scale (HADS) to assess the degree of severity of depressive and anxiety symptoms

Participants will be randomized into three groups via computer system and the intervention provided will be either acceptance and commitment therapy (ACT) and mindfulness-based stress reduction (MBSR). The duration of intervention is 8 weeks.

(1) Acceptance and commitment therapy (ACT): It is a third generation cognitive behavioral approach which uses acceptance and mindfulness processes, and commitment and behavior change processes to produce psychological flexibility. Unlike CBT, which aims to change unhelpful thoughts and feelings, ACT was designed to increase adaptive coping through acceptance, cognitive defusion, mindfulness, and perspective-taking exercises while supporting cancer survivors in aligning behavior with their personal values. Hence, ACT consists of 8 sessions, one session per week to facilitate cancer patients to development and maintenance of health behavioral improvements by targeting internal barriers, such as emotional discomfort and self-defeating thoughts, and by fostering connection and commitment to personal values associated with self-management of positive health behaviors.

(2) Mindfulness-based stress reduction (MBSR): This 8-week programme offer intensive secular mindfulness training to help patient with stress, anxiety, depression and illness. It combined mindfulness meditation, body awareness, yoga and behavioral exploration, thought, emotion and action. The term ‘mindfulness’ is used to refer to psychological awareness, behavior to promote this awareness, that is information processing and role play. The MBSR programme is an 8-week programme involving training in mind meditation and yoga. Patient will meet the therapist once a week. A standard course of MBSR made up of 8 sessions, weekly session of 2.5 to 3 hours, added with a whole day session after the 6th week with full exercise. Each session start with mindfulness exercise learned by the patient during the weekly session. The main exercise for the MBSR programme include body balancing, soft yoga, and sitting meditation. In addition, there will be a lot of centralized training which incorporate mindfulness practice into daily life. Other exercise include mindfulness training which fully focus on thoughts, sensation and emotion. Each session will focus on a particular topic which will be explored with specific exercise as well as therapist presentation and dialogue among the group members. Additional time will be allocated to participants to share what they experience during the exercise and to discuss regarding their mindfulness practices in their daily life outside of the class.

RISKS

Participation in this research has minimal risk. Nevertheless, if you experience emotional disturbance after answering the questionnaires, we will recommend reference to counsellor in Hospital Bukit Mertajam, Pulau Pinang. If you still exhibit depressive and anxiety symptoms after you have completed the study, we will recommend reference to Klinik Minda Sihat, Advanced Medical and Dental Institute, Universiti Sains Malaysia for further assessment and treatment as necessary. Please inform the research team if you encounter any problems or if you have any important information which will change your participation in the study.

REPORTING HEALTH EXPERIENCES.

Please contact, at any time, the following researcher if you experience any health problem either directly or indirectly related to this study.

Dr. Mohammad Farris Iman Leong Bin Abdullah [MMC Registration No. 43103] at 04-5622482 or 018-6669950.

#### PARTICIPATION IN THE STUDY

Your taking part in this study is entirely voluntary. You may refuse to take part in the study or you may stop your participation in the study at anytime, without any penalty or loss of benefits to which you are otherwise entitled. Your participation also may be stopped by the research team without your consent if in any form you have violated the study eligibility criteria. The research team member will discussed with you if the matter arises.

#### POSSIBLE BENEFITS [Benefit to Individual, Community, University]

The research procedures will be provided to you without any cost. You will received information regarding your mental health status and on the positive psychological aspects which are expected to be important to safeguard your mental health after you were diagnosed with cancer and experienced the complications related to it. You will also enrolled in psychotherapy sessions in acceptance and commitment therapy (ACT) or mindfulness-based stress reduction (MBSR) which is expected to help you to maintain your mental health despite having cancer.

The findings of this study will hopefully bring benefits to the community by providing evidence that the two psychotherapy intervention will enhance the positive psychology and quality of life as well as alleviate psychological complications of cancer, such as stigma towards cancer, experiential avoidance, depression, and anxiety. This will allowed recommendation to integrate these two interventions into the treatment regime of head and neck cancer patients as data on the efficacy of these two interventions among head and neck cancer patients is scarce.

You will not recieve any compensation from this study. However you may get reimbursement for your travelling cost while in the study duration.

#### QUESTIONS

If you have any question about this study or your rights, please contact;

Dr. Zhang Zheng

Primary investigator

Department of Community Health

Advanced Medical and Dental Institute

Universiti Sains Malaysia

SAINS@BERTAM

13200 Kepala Batas

Pulau Pinang

04-5622482

Dr. Mohammad Farris Iman Leong Bin Abdullah

Primary investigator

Department of Community Health

Advanced Medical and Dental Institute

Universiti Sains Malaysia

SAINS@BERTAM

13200 Kepala Batas

Pulau Pinang

018-6669950

If you have any questions regarding the Ethical Approval or any issue / problem related to this study, please contact;

Mr. Mohd Bazlan Hafidz Mukrim

Secretary of Human Research Ethics Committee USM

Division of Research & Innovation (R&I)

USM Health Campus

Tel. No. : 09-767 2354 / 09-767 2362

Email : [bazlan@usm.my](mailto:bazlan@usm.my)

OR

Miss Nor Amira Khurshid Ahmed

Secretariat of Human Research Ethics Committee USM

Research Creativity & Management Office (RCMO)

USM Main Campus, Penang

Tel. No. : 04-6536537

Email : [noramira@usm.my](mailto:noramira@usm.my)

#### CONFIDENTIALITY

Your information will be kept confidential by the researchers and will not be made publicly available unless disclosure is required by law.

Data obtained from this study that does not identify you individually will be published for knowledge purposes.

Your original records may be reviewed by the researcher, the Ethical Review Board for this study, and regulatory authorities for the purpose of verifying the study procedures and/or data. Your information may be held and processed on a computer. Only research team members are authorized to access your information. The data will be stored for seven years after completion of the study and then it will be discarded in the standard manner.

By signing this consent form, you authorize the record review, information storage and data process described above.

#### SIGNATURES

To be entered into the study, you or a legal representative must sign and data the signature page [ATTACHMENT S or ATTACHMENT P]

ATTACHMENT S

Subject Information and Consent Form

(Signature Page)

*Research Title: Randomized controlled trial of acceptance and commitment therapy versus mindfulness-based stress reduction in newly diagnosed head and neck cancer patients*

*Researcher’s Name: Dr. Zhang Zheng, Dr. Mohammad Farris Iman Leong Bin Abdullah (MMC: 43103), Dr. Nurul Izzah Shari, Profesor Dr. Lu Ping*

To become a part this study, you or your legal representative must sign this page. By signing this page, I am confirming the following:

- I have read all of the information in this Patient Information and Consent Form including any information regarding the risk in this study and I have had time to think about it.
- All of my questions have been answered to my satisfaction.
- I voluntarily agree to be part of this research study, to follow the study procedures, and to provide necessary information to the doctor, nurses, or other staff members, as requested.
- I may freely choose to stop being a part of this study at anytime.
- I have received a copy of this Participant Information and Consent Form to keep for myself.

Participant Name

Participant I.C No

Signature of Participant or Legal Representative Date (dd/MM/yy)

Name of Individual

Conducting Consent Discussion

Signature of Individual Date (dd/MM/yy)

Conducting Consent Discussion

Name & Signature of Witness Date (dd/MM/yy)

Note: i) All participants who are involved in this study will not be covered by insurance.

ATTACHMENT P

Participant’s Material Publication Consent Form

Signature Page

*Research Title: Randomized controlled trial of acceptance and commitment therapy versus mindfulness-based stress reduction in newly diagnosed head and neck cancer patients*

*Researcher’s Name: Dr. Zhang Zheng, Dr. Mohammad Farris Iman Leong Bin Abdullah (MMC: 43103), Dr. Nurul Izzah Shari, Profesor Dr. Lu Ping*

To become a part this study, you or your legal representative must sign this page.

By signing this page, I am confirming the following:

- I understood that my name will not appear on the materials published and there have been efforts to make sure that the privacy of my name is kept confidential although the confidentiality is not completely guaranteed due to unexpected circumstances.
- I have read the materials or general description of what the material contains and reviewed all photographs and figures in which I am included that could be published.
- I have been offered the opportunity to read the manuscript and to see all materials in which I am included, but have waived my right to do so.
- All the published materials will be shared among the medical practitioners, scientists and journalist world wide.
- The materials will also be used in local publications, book publications and accessed by many local and international doctors world wide.
- I hereby agree and allow the materials to be used in other publications required by other publishers with these conditions:
- The materials will not be used as advertisement purposes nor as packaging materials.
- The materials will not be used out of contex – i.e.: Sample pictures will not be used in an article which is unrelated subject to the picture.

Participant Name

Participant I.C No. Participant’s Signature Date (dd/MM/yy)

Name and Signature of Individual Date (dd/MM/yy)

Conducting Consent Discussion

Note: i) All participants who are involved in this study will not be covered by insurance.
