# Supplementary material for: Acceptance and commitment therapy versus mindfulness-based stress reduction for newly diagnosed head and neck cancer patients: A randomized controlled trial assessing efficacy for positive psychology, depression, anxiety, and quality of life
Source: PLoS One. 2022 May 10;17(5):e0267887. doi: 10.1371/journal.pone.0267887 (PMC9089868; doi:10.1371/journal.pone.0267887)
Supplement: S3 Appendix — (DOCX) [file pone.0267887.s005.docx]

**Randomized controlled trial of acceptance and commitment therapy versus mindfulness-based stress reduction in newly diagnosed head and neck cancer patients**

Mohammad Farris Iman Leong Bin Abdullah^1^ (MMC: 43103) (primary investigator) (GCP certified), Zheng Zhang^1^, Nurul Izzah Shari^1^, Ping Lu^2^

^1^Department of Community Health, Advanced Medical and Dental Institute, Universiti Sains Malaysia, SAINS@BERTAM, 13200 Kepala Batas, Pulau Pinang

^2^Department of Oncology, First Affiliated Hospital, Xinxiang Medical University, Xinxiang, Henan, People’s Republic of China

**Introduction:**

Head and neck cancer is a group of biologically similar cancers that affects oral cavity, lips, nasal cavity, paranasal sinuses, pharynx and larynx. Head and neck cancer affects 550,000 people worldwide annually and leads to 300,000 deaths on a yearly basis (World Health Organization, 2014). In the Malaysian context, 9,419 cases of head and neck cancer were reported between 2007 and 2011, which made up of 9.1% of all cancer diagnoses rendering head and neck cancer as the fourth most prevalent cancer in the country (The Star, 2017). The main differentiating feature-8*96 of head and neck cancer from other types of cancer is the complication of facial disfigurement which may increase the psychological vulnerability of patients due to the society’s emphasis on physical attractiveness. The appearance of facial disfigurement can increase depression and reduced quality of life in head and neck cancer patients (Dropkin 1999; Long et al., 1996). Moreover, head and neck cancer survivors often affected by devastating complications of the cancer itself and the side effects of its treatment, such as fatigue, pain, problem with speech and swallowing, breathing problem, mucositis, xerostomia, and trismus. These often leave the patients with detrimental effects on many functions and activities of daily living which causes further psychological distress and decreasing quality of life (Holtmaat et al., 2017; Sharp et al., 2018). As a result of its high rate of mortality and the hideous facial disfigurement and other complications cause by the cancer itself or due to the adverse effects of its treatment, internalized stigma is often experience by head and neck cancer patients.

**Problem Statement:**

Head and neck cancer is a group of biologically similar cancers which cause deleterious impact. Overall, head and neck cancer affect 550,000 people with 300,000 deaths annually around the globe (World Health Organization, 2014). The main differentiating feature of head and neck cancer from other types of cancer is the complication of facial disfigurement which may increase the psychological vulnerability of patients due to the society’s emphasis on physical attractiveness. The appearance of facial disfigurement can increase depression and reduced quality of life (QoL) in head and neck cancer patients (Hagedoom and Molleman 2006). One important factor which is associated with deterioration of QoL in cancer patients is stigmatization due to diagnosis of cancer. Stigmatization has been reported to reduce all dimensions of QoL in cancer patients (Ernst et al., 2017). Despite being affected by negative complications, such as psychological sequelae and reduced QoL, cancer patients also experienced positive psychology in response to traumatic or significant life events, which enhanced the mental well-being and functioning of cancer patients. Among the positive psychology developed in cancer patients despite their negative experience of cancer and the adverse effects of its treatment are posttraumatic growth (PTG) and hope which may enhance the QoL of cancer patients. Several psychosocial interventions have been suggested to enhance positive psychology in cancer patients and increase in their QoL. Among the psychosocial interventions shown to be promising include mindfulness-based intervention and newer psychosocial intervention, such as acceptance and commitment therapy (ACT). Data is lacking on the efficacy of mindfulness-based stress reduction (MBSR) and acceptance and commitment therapy (ACT) on enhancing positive psychology (such as PTG, optimism and hope) and QoL among head and neck cancer patients. Data on the effect of MBSR and ACT on depression and anxiety, and experiential avoidance among head and neck cancer is even more scarce.

**Hypothesis:**

(1) Head and neck cancer patients in the acceptance and commitment therapy (ACT) group reported significantly increase in posttraumatic growth (PTG), hope, optimism, and quality of life as well as significantly reduced depression and anxiety, and experiential avoidance compared with those in the control group at immediately post-intervention (8 weeks) and 6 months after intervention when compared with pre-intervention.

(2) Head and neck cancer patients in the mindfulness-based stress reduction (MBSR) group reported significantly increase in posttraumatic growth (PTG), hope, optimism, and quality of life as well as significantly reduced depression and anxiety, and experiential avoidance compared with those in the control group at immediately post-intervention (8 weeks) and 6 months after intervention when compared with pre-intervention.

(3) There are no difference in the increase in posttraumatic growth (PTG), hope, optimism and quality of life, and decrease in depression and anxiety, and experiential avoidance between the MBSR and ACT groups at immediately post-intervention (8 weeks) and 6 months after intervention when compared with pre-intervention.

**Research Questions:**

(1) Does acceptance and commitment therapy (ACT) significantly increase in posttraumatic growth (PTG), hope, optimism, and quality of life as well as significantly reduced depression and anxiety, and experiential avoidance compared with those in the control group at immediately post-intervention (8 weeks) and 6 months after intervention when compared with pre-intervention?

(2) Does mindfulness-based stress reduction (MBSR) significantly increase in posttraumatic growth (PTG), hope, optimism, and quality of life as well as significantly reduced depression and anxiety, and experiential avoidance compared with those in the control group at immediately post-intervention (8 weeks) and 6 months after intervention when compared with pre-intervention?

(3) Are there any significant difference in the increase in posttraumatic growth (PTG), hope, optimism and quality of life, and decrease in depression and anxiety, and experiential avoidance between the MBSR and ACT groups at immediately post-intervention (8 weeks) and 6 months after intervention when compared with pre-intervention?

**Literature Reviews:**

***Depression, anxiety, and head and neck cancer patients***

Patients with head and neck cancer has been reported to be prevalent for depression and anxiety due to the complication of facial disfigurement which may increase the psychological vulnerability of the patients due to the society’s emphasis on physical attractiveness (Dropkin, 1999; Long et al., 1996). As a result, it is not surprising that head and neck cancer is associated with psychiatric sequelae such as depression and anxiety. Some studies have found that head and neck cancer has the highest documented rates of depression and anxiety (Strauss, 1989; Massie, 2004). In a study of orofacial cancer patients by Humphris et al. (2003), they found that significant number of patients experienced depression and anxiety 3 months post-treatment (possible case: anxiety= 37% and depression= 28%). While Kugaya et al., 2000, study on head and neck cancer patients using clinical interview with DSM III found that 16.8% of patients have depression.

Depression and anxiety in head and neck cancer could contribute to impaired quality of life of patients (Smith et al., 2003). In an article from Spiegel and Giese-Davis, 2003, they found that there is stronger evidence that depression may predicts cancer progression and mortality, where depression and cancer have a bidirectional relationship; both can exert negative impact on each other. Study in cancer patients has also highlighted that high level of anxiety and depression adversely affects the coping and quality of life of patients (Karakoyun-Celik et al., 2010). Consequently, depression and anxiety in head and neck cancer patients should be adequately treated. It would be interesting to investigate the efficacy of psychosocial interventions focusing on mindfulness and acceptance, such as MBSR and ACT, respectively, on alleviating depressive and anxiety symptoms in head and neck cancer patients.

***Experiential avoidance and head and neck cancer patients***

Experiential avoidance (EA) has been broadly defined as attempts to avoid thoughts, feelings, memories, physical sensations, and other internal experiences—even when doing so creates harm in the long-run (Hayes et al., 1999). The process of EA is thought to be maintained through negative reinforcement—that is, short-term relief of discomfort is achieved through avoidance, thereby increasing the likelihood that the behavior will persist. Now it is believed that negative thoughts, emotions, and sensations do not contribute to psychiatric illnesses, but habitual and persistent unwillingness to experience uncomfortable thoughts and feelings (and the associated avoidance and inhibition of these experiences) is thought to be linked to psychiatric illnesses (Hayes et al., 1996).

In the context of the cancer patients, intervention which successfully induce reduction in experiential avoidance predicted reduction in depressive and anxiety symptoms as well. In addition, reduction in experiential avoidance also indirectly predicted higher quality of life of cancer patients via its effect on depressive symptoms (Aguirre-Camacho et al., 2017). In fact, topic avoidance of talking about cancer among cancer patients resulting in greater level of depression and anxiety. Topic avoidance may decrease use of emotional support and increase self-blaming among the cancer patients, whereby both may lead to higher levels of psychological distress (Donovan-Kicken and Caughlin, 2011). To the best of our knowledge, to date, no study has evaluated EA and psychosocial intervention which could reduce EA among head and neck cancer patients.

***Positive psychology and head and neck cancer patients***

Positive psychology is defined as a scientific approach to study human thoughts, feelings and behaviours which focus on the strength and the good outcomes it brought in life which allow others to progress in life instead of just resulting in one who is struggling in life to improve his/her life up to where it was before the struggle (Peterson, 2008). Positive psychology includes broad types of positive states and traits, such as happiness, gratitude, hope, optimism, self-esteem, self-confidence, well-being, life satisfaction, posttraumatic growth, and benefit finding. One area in life which positive psychology focus on is its effects onto the outcomes of various medical illnesses. Among the positive psychology which may bring about positive outcomes in cancer patients include posttraumatic growth (PTG) and hope.

*Posttraumatic growth and head and neck cancer*

Posttraumatic growth (PTG) is positive psychological changes experienced by a person as a result of struggle due to life-threatening crisis or event. PTG comprised of five components and someone who experienced higher PTG will have greater appreciation of life, improved interpersonal relationship, better personal strength, higher spiritual development and experiencing more possibilities in life. PTG develops only when there is major life crisis or event and it is not develop if someone experienced only minor life event. Instead of acting as a coping mechanism, PTG is an outcome resulting from struggle with the major life event or crisis. PTG develops only when one is attempting to make meaning out of the traumatic major life event. This occurred when the person pre-assumptive world was shattered by the traumatic experience and the person is able to make meaning out of the trauma by incorporating or accommodate the new trauma-related information to rebuild the assumptive world after the traumatic event. Hence, PTG is a phenomenon which resulted in positive psychological changes beyond the level attained prior to the trauma (Tedesche and Calhoun, 2004). PTG is important and should be a positive psychology characteristic to focus on in cancer patients as it is inversely correlated to depression and psychological distress (Shand et al., 2014; Casellas-Grau et al., 2017). It is also positively correlated to health-related quality of life (Tomich and Helgeson, 2012; Casellas-Grau et al., 2017).

Cultural differences may contribute to differences in occurrence of PTG in cancer patients and also influence the factors associated with PTG (Cho and Park, 2013). To date, data is still lacking regarding PTG in Asian head and neck cancer patients. To date, only two studies investigated PTG in Asian head and neck cancer patients. The first study was in Hong Kong oral cavity cancer patients which demonstrated a mean PTGI score of 51.76 (Ho et al., 2011) while the second study was a prospective study in Malaysian head and neck cancer patients which demonstrated a decrease of the PTGI-SF score across time (Leong Abdullah et al., 2015). PTG exhibited by head and neck patients is relatively lower as compared to other types of cancer (Leong Abdullah et al., 2015). This may be due to physical complications of head and neck cancer and its treatment like facial disfigurement, problem with speech and swallowing, xerostomia, and trismus which are not seen in other types of cancer. Hence, knowing that PTG is associated with mental health well-being of head and neck cancer and predicted higher QoL among cancer patients, it is vital to investigate on psychosocial interventions which could effectively enhance PTG in head and neck cancer patients.

*Hope and head and neck cancer patients*

Hope is a positive goal-directed motivational state and it is also a dispositional trait which enables one to have a tendency to adopt a positive outlook in life. It is made up of two components i.e. (a) agency which is the perceived motivation to initiate and sustain movement to achieve goals set and (b) pathway which is perceived ability to generate ways and paths to achieve the goals set. The degree of hope is dependent on how agency interacts with pathway. Hence, agency and pathway must be present in order to increase hope (Snyder et al., 1991).

Hope is associated with several outcomes in cancer patients. Hope is negatively associated with depression, anxiety and psychological distress in cancer patients (Kim et al., 2011; Shimizu et al., 2012; Han et al., 2013; Yang et al., 2014). Hope is also noted to be positively correlated with social connections in which higher hope is associated with better relationship with neighbours and friends, nursing care satisfaction and the quality of the social relationship in cancer patients. Higher hope is associated with better quality of life and spiritual well-being of cancer patients (Jo and Son, 2004; Ryu and Yi, 2013). Higher hope is also positively correlated with greater self-efficacy in cancer patients (Chang and Li, 2002; Lin and Tsay, 2005; Yang et al., 2014). In addition, higher hope is also significantly associated with positive psychology such as posttraumatic growth, optimism, resilience and psychosocial adjustment in cancer patients (Hou et al., 2010; Ho et al., 2011; Ryu and Yi, 2013). In the Malaysian context, hope is reported as the most significant factor associated with greater posttraumatic growth in cancer patients compared with other positive psychology, such as optimism and religious coping (Leong Abdullah et al., 2019). In conclusion, hope is one positive psychology which is important to focus on in cancer patients with particular emphasis place in investigating the psychosocial interventions which may enhance hope; in which data is still lacking to date.

*Optimism and head and neck cancer patients*

Optimism is the stable and consistent believe that good things rather than bad things will happen in one’s life (Scheier et al., 1994). Hence, unlike hope, it is not related to goal-directed motivational state. In fact, studies of cancer patients has demonstrated that optimism and hope are indeed two different parameters (Ho et al., 2011; Leong Bin Abdullah et al., 2019).

But similarly, optimism is associated with several positive outcomes in cancer patients. Optimism is shown to be positively correlated to psychological well-being but inversely correlated to depression and psychological distress. It is also positively associated with health-related quality of life in cancer patients (Miller et al., 1996; Horney et al., 2011; Petersen et al., 2008). In addition, optimism is also positively associated with positive psychology such as posttraumatic growth and hope in cancer patients. Optimism act as a protective cognitive strategy which allow for reappraisal of the traumatic event and hence, it allow one to find meaning in the traumatic event which one experienced (Yi et al., 2015). In the context of head and neck cancer patients, optimism predicts better 1 year survival of head and neck cancer patients independent of confounding demographic and clinical factors (Allison et al., 2003). On the contrary, when the level of optimism in head and neck cancer patients is low, it predicted higher level of depression (Horney et al., 2011).

***Quality of life and head and neck cancer patients***

Quality of life is the perception of one’s position in life in the context of the culture and values of the community one lives in and in relation to standard, goals, concern and expectations (Feelemyer et al., 2014). Quality of life (QoL) is an important measure in the field of psycho-oncology as a health indicator for assessments and treatment outcomes. QoL assessment serves a few purposes in psycho-oncology research, such as an indicator of the therapeutic outcome in the assessment of the efficacy of a treatment for cancer, to assess its association with other variables in the cancer survivors and to be used as a reference tool for the assessment of validity of other instruments for measuring QoL in the cancer patient population (King et al., 2016). Since head and neck cancer is associated with a wide range of illness complications and adverse effects from its treatment which could lead to deterioration in QoL among cancer patients (Holtmaat et al., 2017; Ernst et al., 2017; Sharp et al., 2018), it is pivotal to investigate on psychosocial interventions which could enhance the QoL of head and neck cancer patients. Furthermore, as greater PTG and hope are predictors of higher degree of QoL (Jo and Son, 2004; Casellas-Grau et al., 2017), while internalized stigma is associated with poorer QoL among cancer patients (Ernst et al., 2017), it would be interesting to examine the efficacy of psychosocial interventions which could alter PTG, hope and internalized stigma to enhance the QoL and safeguard the mental health of head and neck cancer.

***Psychosocial interventions for cancer patients***

Psychosocial interventions for mental health are interpersonal or informational activities, techniques, or strategies that target biological, behavioral, cognitive, emotional, interpersonal, social, or environmental factors with the aim of improving health functioning and well-being (Committee on Developing Evidence-Based Standards for Psychosocial Interventions for Mental Disorders et al., 2015). There are a number of psychosocial interventions which have been reported to enhance mental well-being of cancer patients, which can be broadly classified into cognitive behavioral interventions, supportive interventions, group interventions, and telephone-assisted interventions (Raingruber, 2011). Among the psychosocial interventions, we will focus on two interventions which have been reported to enhance positive psychology in cancer patients i.e. acceptance and commitment therapy (ACT) and mindfulness-based stress reduction (MBSR).

*Acceptance and commitment therapy (ACT)*

ACT is a third generation cognitive behavioral approach which uses acceptance and mindfulness processes, and commitment and behavior change processes to produce psychological flexibility. The latter is defined as the ability to contact the present moment more fully as a conscious human being and to change or persist in behavior when doing so serves valued ends (Hayes et al., 1999). Unlike CBT, which aims to change unhelpful thoughts and feelings, ACT was designed to increase adaptive coping through acceptance, cognitive defusion, mindfulness, and perspective-taking exercises while supporting cancer survivors in aligning behavior with their personal values (Johns et al., 2019). The objectives of the therapy are for client to learn that avoidance, suppression or the attempt to control difficult thoughts can be counterproductive. They also learn to focus on behaviors and actions that are in line with their individual values – the things they care about most (Hayes et al., 2006). ACT facilitates development and maintenance of health behavioral improvements by targeting internal barriers, such as emotional discomfort and self-defeating thoughts, and by fostering connection and commitment to personal values associated with self-management of positive health behaviors (Henry et al., 1997). ACT is useful in cancer patients, because it can help them to deal with the negative emotions caused by cancer (e.g. uncertainty, anxiety, sadness and anger) instead of avoiding these. ACT is a 6-week therapy with each session lasted for 2 hours per week.

Emerging evidence has indicated that ACT improve QoL and enhance health behaviors of a number of chronic illnesses, including cancer (Páez et al., 2007; Feros et al., 2011). In a meta-analysis of 25 treatment-control comparison studies with a total sample size of 2256 cancer patients pinpointed that ACT has a large effect size for psychological distress, particularly ACT significantly reduced depression and anxiety symptoms in cancer patients. ACT also had a large effect size to increase QoL of cancer patients and it also exhibited high effect size to increase the degree of hope of cancer patients. Although sample size may be relatively small to justify the large effect size of ACT on the degree of hope, the main principles of ACT which facilitates cancer patients to clarify values and commit action are believed to improve hope in patients. ACT has been reported to benefit younger patients more than older ones which may be reflected by the higher degree of baseline psychological distress and lower baseline degree of acceptance in younger patients which bring about greater degree of increase in acceptance and reduction in psychological distress after ACT was administered (Zhao et al., 2021). Besides, in a randomized control trial of 410 colorectal cancer survivors, ACT has been reported to significantly increase PTG at 6 months and 12 months of telephone-based health coaching intervention using ACT strategies (Hawkes et al., 2014). To date, the effect of ACT on the PTG, hope, and QoL of head and neck cancer patients have not been investigated.

*Mindfulness-based stress reduction (MBSR)*

The term ‘mindfulness’ refers to mindful awareness as a way of being – a knowing and experiencing of feelings, thoughts, and perceptions as they arise and pass away each moment (Shapiro and Carlson, 2009). It is a way of relating to all experiences in an open, receptive way,

without judging experiences as good or bad (grasping at them or pushing them away) (Bishop et al., 2004). Mindfulness meditation, or mindfulness practice, consists of intentionally engaging in the task of focusing and sustaining attention to present-moment experience with

acceptance (Shapiro & Carlson, 2009). Mindfulness employed two styles of meditations i.e. ‘concentrative’ and ‘receptive’ meditation. Concentrative meditation involves intentionally focusing attention on a chosen object (e.g. the sensations of breathing) in a sustained way. Receptive or ‘open awareness’ meditation involves monitoring the content of experience (e.g. sensations, emotions, thoughts, sounds, etc.) in a

nonreactive and non-judgmental way, from moment to moment, with the goal being to recognize the nature of emotional and cognitive patterns. Mindfulness-based stress reduction (MBSR) is an 8-week, standardized group intervention consisting of mindfulness meditation and gentle yoga that is designed to have applications for stress, pain, and illness (Kabat-Zinn, 1990).

Participation of cancer patients in MBSR have been shown to increase spirituality, PTG, self-compassion, and positive states of mind

(Birnie et al., 2010; Bränström et al., 2010; Carmody and Baer, 2008; Garland et al., 2007; Kvillemo and Bränström, 2010; Lengacher et al., 2009; Mackenzie et al., 2007). Studies have highlighted that MBSR increase PTG among cancer patients who participated immediately after the end of the 8-week intervention (Garland et al., 2007; Mackenzie et al., 2007; Labelle et al., 2015). A meta-analysis of 29 studies with a total of 3476 cancer survivors to evaluate the efficacy of mindfulness-based interventions on facilitating mental well-being revealed that MBSR significantly lower depression, anxiety, stress, and fatigue, while it enhances PTG, QoL and mindfulness among cancer patients (Ng et al., 2020). Moreover, MBSR has been reported to lower psychological distress and improve total, social and emotional QoL among head and neck cancer (Pollard et al., 2017). To date, the effect of MBSR on the degree of hope in cancer patients has not been investigated. Hence, it would be interesting to investigate the effect of MBSR on the PTG, hope, and QoL of head and neck cancer patients.

***Study rationale***

As head and neck cancer differ from other types of cancer due to the complication of facial disfigurement which may increase the psychological vulnerability of patients due to the society’s emphasis on physical attractiveness. Moreover, a number of devastating complications of the cancer itself and the side effects of its treatment such as fatigue, pain, problem with speech and swallowing, breathing problem, mucositis, xerostomia, and trismus which further exerts detrimental effects on many functions and activities of daily living which causes further psychological distress and decreasing quality of life. Hence, it is of utmost importance to investigate on psychosocial interventions which could enhance PTG, hope, optimism and QoL as well as reducing internalized stigma, depression and anxiety, and experiential avoidance of head and neck cancer patients which in turn bring about the ultimate outcome of restoring mental and physical well-being of the cancer survivors. Acceptance and commitment therapy (ACT) and mindfulness-based stress reduction (MBSR) have been reported to alleviate positive psychology and reduced psychological distress in cancer patients. But the effect of ACT and MBSR on PTG, hope, optimism, QoL, depression and anxiety, and experiential avoidance among head and neck cancer patients have not been studied. Hence, there is a need to conduct a 3-armed randomized control trial to evaluate the effects of ACT and MBSR on PTG, hope, optimism, QoL, depression and anxiety, and experiential avoidance compared with control group with no intervention across time.

Newly diagnosed head and neck cancer patients

**Compare with**

Acceptance and commitment therapy (ACT)

**Compare with**

Control group with no intervention

Mindfulness-based stress reduction (MBSR)

Assessment of posttraumatic growth, hope, optimism, internalized stigma, depression, anxiety, experiential avoidance and quality of life across time

Increase posttraumatic growth, hope, optimism, quality of life and reduce internalized stigma, depression, anxiety, and experiential avoidance across time in the ACT and MBSR groups

**Improve mental and physical well-being of head and neck cancer patients**

**Figure 1. Conceptual framework of the study**

**Figure 1. Conceptual framework of the study**

**Objective (s) of the Research:**

***General objective:***

To examine the effects of acceptance and commitment therapy (ACT) and mindfulness-based stress reduction (MBSR) on posttraumatic growth (PTG), hope, optimism, quality of life, depression, anxiety, and experiential avoidance among head and neck cancer patients.

***Specific objectives:***

(1) To examine the changes in the degree of posttraumatic growth (PTG), hope, optimism, quality of life, depression, anxiety, and experiential avoidance of head and neck cancer patients in the acceptance and commitment therapy (ACT) group compared with those in the control group at immediately post-intervention (8 weeks) and 6 months after intervention compared with pre-intervention.

(2) To examine the changes in the degree of posttraumatic growth (PTG), hope, optimism, quality of life, depression, anxiety, and experiential avoidance of head and neck cancer patients in the mindfulness-based stress reduction (MBSR) group compared with those in the control group at immediately post-intervention (8 weeks) and 6 months after intervention compared with pre-intervention.

(3) To determine whether there are any difference in the changes in the degree of posttraumatic growth (PTG), hope, optimism, quality of life, depression, anxiety, and experiential avoidance between those in the acceptance and commitment therapy (ACT) group and mindfulness-based stress reduction (MBSR) group at immediately post-intervention (8 weeks) and 6 months after intervention compared with pre-intervention.

**Description of Methodology:**

**Methodology:**

***Study Setting:***

The study will be conducted the Oncology and Otorhinolaryngology unit of Advanced Medical and Dental Institute, Universiti Sains Malaysia, and Department of Oncology, Otorhinolaryngology and Oral and Maxillofacial Surgery of Universiti Kebangsaan Malaysia Medical Centre (UKMMC). The Oncological and Otorhinolaryngology units of AMDI, USM have about 200-250 registered head and neck cancer patients currently under follow up. These units receive new cases of head and neck cancer every week. While the Department of Otorhinolaryngology and Department of Oncology of UKMMC have an estimated 350-400 registered head and neck cancer patients while the Department of Oral and Maxillofacial Surgery of UKMMC has an estimated 100-150 registered oral cancer patients under follow up (oral cancer is grouped under head and neck cancer).

***Study design:***

This study is foreseen in 2021 for an overall period of 3 years from 1st July 2021 to 30^th^ June 2022. This study is a multicentre 3-armed longitudinal double blind randomized control trial which is expected to run for a duration of 2_1/2_ years (from January 2022 to June 2024).

**Sample size:**

The sample size is determined based on G-Power 3.1.9.2 for repeated measures, between-within interaction ANOVA. Based on the previous study, the sample size was calculated based on the continuous response, Posttraumatic growth inventory from the study conducted by Labelle et al. (2014), with small to medium effect size (0.155), an alpha of 0.05, two-tailed. The results indicated that the total sample of 87 for three equal-sized groups is needed to achieve a power of 0.95. In anticipation of a drop-out rate of 30%, the estimation for sample size is 113 respondents and hence, we round up to 120 subjects for the total respondents needed. Therefore, 40 respondents for each group.

***Sampling method:***

The sampling method use in this study for recruitment of participants is by consecutive sampling.

***Recruitment of subjects:***

The participants were recruited from the source population which included all newly diagnosed head and neck cancer patients who has been treated only with surgery or still remain untreated registered under the Oncology and Otorhinolaryngology unit of Advanced Medical and Dental Institute, Universiti Sains Malaysia, and the Department of Oncology, Otorhinolaryngology and Oral and Maxillofacial Surgery of Universiti Kebangsaan Malaysia Medical Centre. These patients will be approached by the research team and explained on the study objectives and procedures. To ensure that the selected participants attended all the sessions in the intervention groups, we will approach those patients who are about to enter for radiotherapy and adjuvant chemotherapy. Those who are interested to participate in the study will be screened for inclusion and exclusion criteria.

The inclusion criteria are:

(i) Those who are newly diagnosed with primary head and neck cancer confirmed by histopathological examination report and at any stage of cancer

(ii) Age 18 years and above.

(iii) Can understand and write in English or Malay.

(iv) Those who are post-surgery and plan for the standard regime of radiotherapy and adjuvant chemotherapy.

(v) Those who developed depression and anxiety symptoms after diagnosis with head and neck cancer with Hospital Anxiety and Depression Scale (HADS)- Depression subscale score of ≥ 8 and HADS- Anxiety subscale score of ≥ 8.

The exclusion criteria are:

(i) Those with history of pre-existing psychiatric illnesses, such as schizophrenia, schizophreniform disorder, schizoaffective disorder, delusional disorder, brief psychotic disorder, bipolar mood disorder, posttraumatic stress disorder, obsessive compulsive disorder, and neurodevelopmental disorders (patients are screened with Mini International Neuropsychiatric Interview to exclude these disorders).

(ii) Those with history of illicit drug intake and those with substance use disorder, alcohol use disorder, substance-related disorders and alcohol-related disorders (patients are screened with urine dipstick for drugs and Mini International Neuropsychiatric Interview to exclude these conditions).

(iii) Those with history of medical illnesses which can induced psychiatric symptoms such as cancer, stroke, coronary heart disease, congestive cardiac failure, COPD, bronchial asthma, systemic lupus erythematosus, rheumatoid arthritis, renal failure, hepatic failure, endocrine disorders, multiple sclerosis, Parkinson’s Disease, and epilepsy.

(iv) Those who are currently on any psychotherapy or counselling sessions.

(v) Those who are physically unfit to perform intervention.

(vi) Those who exhibit cognitive impairment (patients are screen with Mini Mental State Examination, in which those with score of < 24/30 will be excluded).

Those who fulfilled all inclusion criteria without any exclusion criteria will be offered to participate in the study where they are given thorough explanation about the study by the research team:

(i) the participation in the study is voluntary and if they decide to take part, they can withdraw at any time without giving any reason and without loss of any benefit they were entitled for prior to the study.

(ii) the description of the study, the procedure and the assessments involved in the study.

(iii) the safe storage of data

(iv) the implications of the study.

(v) the possible use of data for publication and consent to use the data for policy planning purposes.

After explaining about the study, if the subject verbally agreed to participate in the study, they are given the participant information sheet for further reading and the study participation consent forms to sign. They are enrolled in the study after signing the study participation consent forms.

***Randomization***

The randomization method use for this study is stratified permuted block randomization, in which the trial participants is stratified according to age (the strata selected are 18 to 40 years old, 41 to 60 years old, and more than 60 years old) and gender (the strata are male and female). Participants will be randomized into three groups, such as acceptance and commitment therapy (ACT) group, mindfulness-based stress reduction (MBSR) group and control group on waiting list. The participants will be randomized into one of the three groups in a 1:1:1 allocation ratio by block randomization. The allocation sequence is concealed in opaque, sequential numbered envelope.

***Data collection:***

Data collection is carried out by a research assistant who is not involved in conduct of the study and data analysis. In the pre-intervention phase (T1), the participants in all three groups are administered the following questionnaires:

(i) Socio-demographic and clinical questionnaire which includes age, gender, marital status, employment, education, monthly income, religion, stage of cancer and type of head and neck cancer.

(ii) The Posttraumatic Growth Inventory-Short Form (PTGI-SF) to assess the degree of PTG of the participants

(iii) The Dispositional Hope Scale to assess the degree of hope of the participants

(iv) The Life Orientation Test-Revised (LOT-R) to assess the degree of optimism

(v) The Acceptance and Action Questionnaire (AAQ-II) to assess the degree of experiential avoidance

(vi) The Functional Assessment of Cancer Therapy – Head & Neck (FACT-H & N) to assess the degree of quality of life

(vii) The Hospital Anxiety and Depression Scale (HADS) to assess the degree of severity of depressive and anxiety symptoms

Then the assessments with the following questionnaires are repeated immediately post-intervention (T2) and 6 months after intervention (T3) among participants in all the three groups:

(i) The Posttraumatic Growth Inventory-Short Form (PTGI-SF) to assess the degree of PTG of the participants

(ii) The Dispositional Hope Scale to assess the degree of hope of the participants

(iii) The Life Orientation Test-Revised (LOT-R) to assess the degree of optimism

(iv) The Acceptance and Action Questionnaire (AAQ-II) to assess the degree of experiential avoidance

(v) The Functional Assessment of Cancer Therapy – Head & Neck (FACT-H & N) to assess the degree of quality of life

(vi) The Hospital Anxiety and Depression Scale (HADS) to assess the degree of severity of depressive and anxiety symptoms

***Blinding***

The participants will be blinded for the study as randomization into designated groups are conducted by a research assistant not involved in the study and the allocation is concealed in opaque, sequential numbered envelope. Therefore, the participants will not know which group they are allocated to. Although participants in the ACT and MBSR groups undergo psychosocial intervention earlier, the participants in the control group (who are assigned in the waitlist) will also undergo ACT once they have completed assessments at three-time frames (T1, T2, and T3). Participants will not be informed about when they will receive the intervention programme in order to assure blinding. All the participants will also be blinded regarding the hypotheses of the study.

The researchers will be blinded for the study as randomization of participants into designated groups are conducted by a research assistant not involved in the conducting the study and data analysis. Data collection will also be caried out by the research assistant who is not involved in conducting the study and data analysis and blinded regarding the hypotheses of the study. Moreover, the data analysis will be performed out by statisticians who are not involved in conducting the study and blinded regarding the hypotheses of the study.

Approach eligible patients

Consenting patients (n=120)

**Enrolment**

Identification of eligible patients

Measures for pre-intervention at T1

AAQ II, PTGI-SF, SSS, HOPE, LOT-R,

FACT-H & N, HADS

Waitlist (n=40)

MBSR intervention (n=40)

MBSR session 1

ACT intervention (n=40)

ACT session 1

**Allocation**

Standard care

MBSR session 2

ACT session 2

MBSR session 3

ACT session 3

MBSR session 4

ACT session 4

Measures for post-intervention at T2

AAQ II, PTGI-SF, SSS, HOPE, LOT-R,

FACT-H & N, HADS

MBSR session 5

ACT session 5

**Data collection**

MBSR session 6

ACT session 6

MBSR session 7

ACT session 7

Measures for follow-up at T3

AAQ II, PTGI-SF, SSS, HOPE, LOT-R,

FACT-H & N, HADS

MBSR session 8

ACT session 8

Follow-up (6 month after completed intervention)

**Figure 2. The study procedures**

***Interventions:***

Both ACT and MBSR intervention will be conducted in a group of 10 for each session. The ACT and MBSR modules covered over 8 sessions, 1 hour in each session. The sessions will be held every week according to the patient's appointment date for chemotherapy treatment.

(1) Acceptance and commitment therapy (ACT):

*Session 1:* This session will be started with developing a therapeutic relationship and an initial intake interview with respondents. The personal information of respondents such as the main external barrier, fusion past, experiential avoidance, unworkable action, strengths, resources and past life history will be gathered. Based on the information given, the therapist will generate a case formulation. Then, the case formulation will be used in mapping the respondents' life issues on a matric diagram.

*Session 2:* The therapist will start the session by discussing the issues from the previous session and homework. Then, the respondents will be encouraged to embrace their unpleasant feelings and thoughts (creative hopelessness). The therapist will introduce the new possibilities such as the unpleasant feelings and thoughts are not the enemy, but control is the problem. The alternative to control is a willingness to give space and “sit” with the unpleasant thought, feeling, and memory. Then, the respondents are encouraged to actively “contact” with their psychological experiences without struggling, “sit with” and make space for their experiences and remain at the present moment. Several metaphors will be used such as tug of war with the monster and physicalizing the unpleasant thoughts and feelings. The metaphors will help the respondents to recognize their experiential avoidance and “let go” of unhelpful emotion-control strategies.

*Session 3:* In this session, the therapist will conduct a defusion session with the respondents. Defusion will help to reduce behavioural avoidance from unpleasant emotions and thoughts. Defusion is aimed to increase the extent to which respondents’ abilities to choose actions based on their values rather than being stuck and controlled by their emotions and thoughts. The therapist will use metaphors such as hands trap, the mind is a bully and passengers on the bus and performs pushing paper exercise with respondents. As for homework, the therapist will encourage respondents to do physicalizing exercises and passengers on the bus when they deal with their unwanted thoughts and feelings.

*Session 4:* In this session, the therapist will guide the respondents to practice structured mindfulness exercises namely mindful breathing. In the mindfulness exercise, the therapist will encourage the respondents to focus on the present moment, for example in breathing, the respondents are encouraged to use their senses during breathing by focusing on inhaling and exhaling, noticing the temperature of the air, and movement of their muscles during breath in and out. Then, awareness will be expanded by noticing the external environment such as sights and sounds from her surrounding, sensations of body contact and body posture. After that, the respondents will be asked to do mindful breathing while simultaneously acknowledge the presence of their unpleasant thoughts and feelings. The therapist will encourage the respondents to experience the thoughts and feeling with openness, interest and receptiveness without attempted to change them. Instead of trying to distract respondents from their unpleasant feelings and thoughts, the purpose of this activity was to awake respondents that there are many more things that happened right now that can be appreciated and enjoyed rather than overly focused on unpleasant feelings and thoughts. When the respondents become more aware, they could respond efficiently, particularly when experiencing unpleasant feelings and thoughts. The therapist will use the metaphor of an emotional storm and dropping anchor. In this metaphor, the therapist emphasizes that dropping an anchor does not make the storm leave but it hold the boat firmly during the storm. The storm will come and go in its own time. As homework, the respondents will be encouraged to practice mindfulness. At least, mindful breathing twice daily, after wake-up in the morning and before goes to sleep. The respondents also need to record mindfulness exercises in the mindfulness worksheet.

*Session 5:* In this session, the therapist will check the mindfulness exercise worksheet and ask respondents’ experience in practicing mindfulness. Then, the therapist also encouraged the respondent to use mindfulness in her daily activities such as mindful eating, and mindful bathing.

*Session 6:* This session is aimed to develop respondents’ sense of self as context. The respondents will learn that the self is safe, continuous, consistent and separate from their unpleasant thoughts and feelings. The respondents will be asked to develop a position from which they can observe their unpleasant thoughts and feelings and let them come and go. Again, the therapist will use metaphors such as chessboard and furnished room to ensure that respondents understand the concept of self as context.

*Session 7:* This session involved therapeutic processes in hexaflex namely values. This session is aimed to ensure that respondents have clearly expressed their values. The therapist will help the respondents to clarify their values and identify what important in their life such as relationships, health, education and spirituality. Values are the anticipated qualities of ongoing action, freely chosen and never need to be evaluated. Values differ from goals. Once values are identified, then, goals can be established. Some exercises such as values assessment, values compass and bull eye were conducted to help the respondents differentiate between values and goals, identify and evaluated whether their life engaged with their values or not.

*Session 8:* In this session, the therapist will discuss again the values determined by the respondent from the previous session. This session aims to ensure that respondents will be able to link their values to action strategies. The therapist will ask respondents to list the feasible actions and strategies. Finally, the therapist will encourage the respondents to take at least minimal steps or actions that could move in the direction of their valued life.

(2) Mindfulness-based stress reduction (MBSR):

*Session 1:* In this session, the therapist will discuss and explain to respondents about mindfulness-based therapy and the importance of home practice. Spending time to do home practice is a challenging part of this course but the outcomes are worth doing. Then, the therapist will do the automatic pilot activity. This activity aims to increase awareness, then, we can respond to the situation by choice and not react automatically. In the automatic pilot, we tend to dimly aware of what we are doing and follow habits of thinking which are unhelpful and lead to stress. The therapist will do a ‘raisin exercise’ to show how our attention is not always placed fully in the present moment. Then, the therapist will emphasize that when become more aware of the present moment, our thoughts feelings and bodily sensations, we can give ourselves greater freedom and choice. Mindfulness is not about trying to get anywhere but being aware of where and how we are allowing ourselves to be that. At the end of the session, the therapist will introduce respondents to the body scan exercise. In the body scan exercise, respondents are encouraged to place attention on the different parts of the body and use each part of our body as the anchor of our awareness at the moment. Before the end of the session, the therapist will ask respondents to do a body scan 6 times at home and listening to the mindfulness CD.

*Session 2:* In this session, the therapist reviews the previous session lessons and home practices. The therapist will discuss the body scan exercise, practicing meditation techniques, practicing mindfulness breathing meditation technique with the respondents, and giving assignments to the respondents.

*Session 3:* In this session, the therapist will review the assignments and previous session lessons, practicing sitting meditations and taking feedback from respondents, doing 3-min breathing exercises, and giving assignments to the respondents.

*Session 4:* In this session, the therapist will review the home practices, doing 5-min exercises of seeing or hearing, and practicing mindfulness of the body in movement and mindful stretching. Then, the therapist will explain that the bodily discomfort we almost inevitably encounter when practice mindful stretching provides an ideal situation to learn how to approach the difficult and unwanted with curiosity, gentleness, kindness and courage. Practice the breathing space together with respondents. Home practice for this week is to practice mindful movement by using the CD track daily for 6 days.

*Session 5:* The therapist will start the session by reviewing the assignments and lessons learned in the previous session. Then, practicing standing yoga, and sitting meditation. After that, the therapist will discuss how to respond instead of reacting. In MBSR, the core skill is learning to replace unconscious stress-reaction with conscious stress responses. For example, when respondents practicing sitting meditation regularly, it will help us to notice when our awareness will drift away and how to gently bring back our attention. We will become more aware so that we can respond mindfully rather than react automatically.

*Session 6:* in this session, the therapist will discuss the previous lesson and emphasize respondents to use our body as a way to awareness. By remaining aware of the present moment, we can mindfully respond instead of automatically react to the stressor. However, to do that we need to experience the stressor just as it is in the present moment, accept and let it be. Then, do mindful walking and 3 minutes breathing space exercise.

*Session 7:* Discuss lessons learned from the previous session; assessing sleep, quality of life, and mental health including unwanted thoughts and feelings. Then, the therapist will discuss that the thoughts are not facts but are mental events. The therapist will ask respondents to deal with unwanted thoughts with breathing space.

*Session 8:* In the last session, the therapist will summarize the content of previous sessions, and discussing programs and encourage respondents to continue mindful exercises regularly. After reconnecting with expanded awareness, the therapist also will encourage respondents to take some considered action in dealing with troubling feelings such as do some pleasurable activities or do something that will give them a sense of satisfaction.

Therapists

The trainee therapists are two post-graduate students who enrolled in their Doctor of Philosophy (Ph.D.) in psychology. They received training approximately 16 hours of ACT intervention and 16 hours of MBSR intervention. In ACT intervention training, trainee therapists will learn about general core concepts of ACT, principals, model, framework, and philosophy. Besides, they also will learn how to apply ACT therapy including engagement with each element in hexaflex, integrating exercises, metaphors, techniques, and mindfulness in ACT sessions and clinical practices. In the MBSR training, the trainee therapists will learn the basic overview and concepts of MBSR including mindfulness, body scan and yoga. Besides, they also will experiential learning about formal and informal practices, recording and home practices.

Control group

Participants randomized to the control group will be in the waiting list for therapeutic intervention which will be administered after they completed the study. They will be provided with supportive needs while awaiting therapeutic intervention after the end of the study. Those who still exhibit any emotional disturbance will be given rescue medication (benzodiazepine) to ease their symptoms on when necessary basis.

***Research tools:***

(1) MINI International Neuropsychiatric Interview:

MINI is a brief structured interview for the major Axis I psychiatric disorders in DSM-IV and ICD-10 to explore 17 disorders. It was designed to meet the need for short but accurate structured psychiatric interview for multi-centre clinical trials and epidemiology studies. It has an administration time of 15 minutes. As compared with SCID-P for DSM-III-R and the CIDI (a structured interview developed by the World Health Organization for lay interviewers for ICD-10), MINI has acceptably high validation and reliability scores, but can be administered in a much shorter period of time (mean 18.7 ±11.6 minutes, median 15 minutes). It can be used by clinicians after a brief training session. Lay interviewers require more extensive training (Sheehan et al., 1998). MINI has good inter-rater and test-retest reliability and the kappa coefficient, sensitivity and specificity were good and very good for all diagnoses except for GAD (kappa= 0.36), agoraphobia (kappa= 0.59) and bulimia (kappa= 0.53) (Lecrubier et al., 1997). The Malay version of MINI was validated and shown to have good psychometric properties. The inter-rater reliability was satisfactory (0.67 to 0.85) and the concordance between the Malay version of the MINI’s and expert diagnoses was good with kappa values greater than 0.88. The Malay version of MINI also adjusted to clinical setting and for assessment of positive cases in the community (Mukhtar et al., 2012). This tool is used in this study to exclude head and neck cancer patients with pre-existing psychiatric illnesses from the study.

(2) Mini Mental State Examination (MMSE):

MMSE is a structured questionnaire comprised of 30 points used to measure cognitive impairment and gauge its severity and progression in clinical and research settings. It has administration time of 5-10 minutes and examine cognitive functions such as time and place

orientation, registration, attention and concentration, recall, language, repetition and complex commands (Pangman et al., 2000; Tuijl et al., 2012). It is sensitive and has good reliability and validity. A score of 24/30 or higher indicate normal, 19-23/30 indicate mild cognitive impairment, 10-18/30 is moderate while 9/30 or lower is severe cognitive impairment. Malay version of MMSE has also been validated for use in Malaysian population (Ibrahim et al., 2009). In this study, it is used to assess cognitive function of patients to ensure that those selected are fit to answer all the questionnaires.

(3) Posttraumatic Growth Inventory-Short Form:

Posttraumatic Growth Inventory is an instrument used to assess the experience of positive change in a person that occurs as a result of traumatic events experienced by the person. The scale can be divided into 5 factors which are personal strength, spiritual change, new

possibilities in life, appreciation of life and relating to others. Each item rated with score range from 0 (I did not experience this change) to 5 (I experienced this change to a great degree) (Tedeschi and Calhoun, 1996). Posttraumatic Growth Inventory-Short Form (PTGI-

SF) is used in this study which is the shorter version of the original PTGI and consists of 10 items where each of the 5 factors in

posttraumatic growth is measured by 2 items. The higher the PTGI-SF score, the higher the level of posttraumatic growth in the individual being assessed. PTGI could be substituted by PTGI-SF with little loss of information (Cann et al., 2010). The PTGI-SF Malay version was translated and back translated independently by language experts from School of Language and Literacy, USM and content validity confirmed by a group of 2 psychiatrists and one posttraumatic growth expert from UKMMC. Its internal consistency demonstrated Cronbach’s alpha of 0.887 with the Cronbach’s alpha of the 5 factors ranging from 0.700 to 0.813. Intraclass correlation coefficient (ICC) gave a good score of 0.75 (95% CI: 0.67-0.81). Confirmatory factor analysis demonstrated 5 factors in which there are 2 items for each factor as in the original version of PTGI-SF (Leong Bin Abdullah et al., 2017a). In this study, it is used to assess level of posttraumatic growth among the participants.

(4) Dispositional Hope Scale:

It is a self-rated 12 items scale which assesses the responder’s level of hope. It comprised of 2 subscales which incorporates Snyder’s cognitive model of hope i.e. (a) Agency (goal-directed energy) and (b) Pathways (planning to accomplish goals). 4 of the 12 items assess agency while another 4 items assess pathways. The other 4 items are fillers. Each item is scored using Likert-point scale from Definitely False to Definitely True (Everson et al., 1996). The Malay version of Hope Scale was translated and back translated independently by language experts from School of Language and Literacy, USM and content validity confirmed by a group of 2 psychiatrists and a clinical psychologist from UKMMC. Its internal consistency demonstrated Cronbach’s alpha of 0.716. Intraclass correlation coefficient (ICC) gave a good score of 0.67 (95% CI: 0.57-

0.75). Confirmatory factor analysis demonstrated 2 factors in which there are 4 items for each factor as in the original version of Hope Scale (Leong Bin Abdullah et al., 2018). In this study, it is used to assess level of hope among the participants.

(5) The Functional Assessment of Cancer Therapy – Head & Neck (FACT-H & N):

Functional Assessment of Cancer Therapy – Head & Neck (FACT-H & N) is a self-reported tool which measures quality of life in patients with head and neck cancer. FACT-HN consists of 39 items and consists of 5 subscales: physical well-being (7 items), social/family well-being (7 items), emotional well-being (6 items), functional well-being (7 items) and head & neck cancer additional concerns (12 items). Each item is scored in a 5-point Likert scale ranging from 0= Not at all to 4= Very much. The higher the score, the greater is the degree of QoL. The tool also registered excellent psychometric properties (Cella et al., 1993). The FACT-H & N has been translated and validated in the Malaysian cancer population. All the subscales have moderate to good internal consistency with Cronbach’s α ranging from 0.65 to 0.87 (Doss et al., 2011). In this study, it is used to assess level of QoL among the participants.

(6) Life Orientation Test-Revised (LOT-R):

LOT assess optimism and pessimism. LOT consist of 8 items plus fillers. Half of these items framed in optimistic manner and another

half in pessimistic manner, and the responders extend their agreement or disagreement in a multipoint scale for each item. It has good psychometric properties but was criticize because the optimistic and pessimistic item set form 2 factors that are not always interrelated. Thus, LOT-R was introduced which is more brief (6 coded items with 3 framed in each direction). The revision omitted or rewrote items that did not focus on explicit expectations. It has good internal consistency and stable over time. The positive and negative subsets are more strongly related to each other than those in LOT (Scheier et al., 1994). The Malay version of LOT-R was translated and back translated independently by language experts from School of Language and Literacy, USM and content validity confirmed by a group of 2 psychiatrists and a clinical psychologist from UKMMC. Intraclass correlation coefficient (ICC) gave a good score of 0.62 (95% CI: 0.50-0.71). Confirmatory factor analysis demonstrated 2 factors in which there are 3 items for each factor as in the original version of LOT-R with factor loading ranging from 0.46 to 0.72 (Leong Bin Abdullah et al., 2017b). In this study, it is used to assess the level of optimism of the participants.

(7) The Acceptance and Action Questionnaire (AAQ-II):

Acceptance and Action Questionnaire (AAQ-II) measured experiential avoidance or psychological inflexibility. AAQ II is the second version and revised from the original version which consists of a shorter version (7 items) with better psychometric consistency. The scores were calculated by summing up the seven items. Higher scores indicated higher levels of psychological inflexibility (Bond et al., 2011). Validation of the Malay version of the AAQ-II indicated that the tool had excellent internal consistency with Cronbach’s α of 0.91 and was a unidimensional scale that measured psychological inflexibility/experiential avoidance (Shari et al., 2019). In this study, it is used to assess the degree experiential avoidance in the participants.

(8) The Hospital Anxiety and Depression Scale (HADS):

The HADS is a self-rated questionnaire that assesses patients’ severity of anxiety and depression using seven items designated for depression subscale and seven items for anxiety subscale. It is suitable for use in assessing severity of depression and anxiety symptoms in patients with medical illnesses, such as cancer patients as HADS focus on the psychological symptoms rather than on physical symptoms in which the latter may be present in cancer as well as depression and anxiety. Each item scores from 0 to 3, and the range of total score for both depressive and anxiety subscales ranged from 0 to 21 per subscale (Zigmond et al., 1983). The cut-off for caseness of depression is 8/21 and the cut-off for caseness of anxiety is also 8/21. The anxiety subscale has a sensitivity of 0.9 and specificity of 0.78 and the depression subscale has a sensitivity of 0.83 and specificity of 0.79 (Bjelland et al., 2002). The Malay version of HADS has been validated in Malaysian breast cancer patients and exhibited acceptable to good internal consistency for its total score and subscales with Cronbach’s α ranging from 0.73 to 0.87 (Yong et al., 2016).

***Data analysis:***

Data analysis will be performed using SPSS version 26 software. Descriptive statistics will be used to analyze demographic and clinical data. In order to achieve objective (1) of the study, the internal consistency, convergent validity, discriminant validity and construct validity (exploratory and confirmatory factor analysis) of the Malay version of the SSS will be evaluated.

Descriptive statistics for demographic and clinical characteristics, and the PTG, hope, optimism, QoL, internalized stigma, depression, anxiety and experiential avoidance scores will be computed. All categorical variables will be presented in frequency and percentage. While all continuous variables will be reported in mean and standard deviation. In addition, inference analyses will be used to evaluate the significant difference between intervention and waitlist groups (categorical variables compared with Pearson’s chi square test, while continuous variables compared with one-way ANOVA). In order to achieve objectives (2) to (4), that is to identify the efficacy of acceptance and commitment therapy (ACT) and mindfulness-based stress reduction (MBSR) on the measured variables, a few tests will be carried out. The comparison of mean differences pre-intervention (T1), post-treatment (T2) and 6 months after intervention (T3) will be examined to determine whether any changes in the measured variables (PTG, hope, optimism, QoL, internalized stigma, depression, anxiety and experiential avoidance) in the ACT, MBSR and control groups across the three timelines by using two-way repeated measure ANOVA. The effect sizes will be calculated to determine how substantially patients' perception towards measured variables changed with and without ACT and MBSR interventions. Assessments will be performed according to the intention-to-treat principle. Statistical significance is set at p < 0.05 and two-tailed.

**Expected results:**

Table 1. Socio-demographic and clinical characteristics

| Variables | ACT group | | MBSR group | | Control group | |
| --- | --- | --- | --- | --- | --- | --- |
|  | Frequency (n) | Percentage (%) | Frequency (n) | Percentage (%) | Frequency (n) | Percentage (%) |
| Gender:  Male  Female |  |  |  |  |  |  |
| Age:  18 to 60 years  > 60 years |  |  |  |  |  |  |
| Religion:  Islam  Other religions |  |  |  |  |  |  |
| Education:  Up to secondary education  Up to tertiary education |  |  |  |  |  |  |
| Diagnosis:  Nasopharyngeal carcinoma  Other types of head and neck cancer |  |  |  |  |  |  |
| Stage of cancer:  Stage 1  Stage 2  Stage 3  Stage 4 |  |  |  |  |  |  |
| Mode of cancer treatment:  Surgery and adjunctive radiotherapy  Surgery and adjunctive chemotherapy  Neoadjuvant chemotherapy and surgery |  |  |  |  |  |  |

Table 2. Comparison of the changes in the degree of posttraumatic growth (PTG), hope, optimism, quality of life, internalized stigma, depression, anxiety, and experiential avoidance between those in the acceptance and commitment therapy (ACT) group and mindfulness-based stress reduction (MBSR) group across time

| **Variables** | **Time 1 (pre-intervention)** | | | **Time 2 (immediately post-intervention)** | | | **Time 3 (6 months after intervention)** | | | **p-value** | **Effect size** |
| --- | --- | --- | --- | --- | --- | --- | --- | --- | --- | --- | --- |
|  | ACT | MBSR | CG | ACT | MBSR | CG | ACT | MBSR | CG |  |  |
| Hope |  |  |  |  |  |  |  |  |  |  |  |
| Optimism |  |  |  |  |  |  |  |  |  |  |  |
| Quality of life |  |  |  |  |  |  |  |  |  |  |  |
| Depression |  |  |  |  |  |  |  |  |  |  |  |
| Anxiety |  |  |  |  |  |  |  |  |  |  |  |
| Experiential avoidance |  |  |  |  |  |  |  |  |  |  |  |
| Posttraumatic growth |  |  |  |  |  |  |  |  |  |  |  |

* statistical significance at p < 0.05, ACT= acceptance and commitment therapy group, MBSR= mindfulness-based stress reduction, CG= waitlist control group

Newly diagnosed head and neck cancer patients registered in Oncology and Otorhinolaryngology unit of Advanced Medical and Dental Institute, Universiti Sains Malaysia, and Department of Oncology, Otorhinolaryngology and Oral and Maxillofacial Surgery of Universiti Kebangsaan Malaysia Medical Centre

Potential subjects briefly explained about the study

Participant screen for inclusion and exclusion criteria with face-to-face interview and Mini International Neuropsychiatric Interview by the research team

Those who fulfilled all inclusion criteria with no exclusion criteria

Those with exclusion criteria

They are excluded from study

Participant signed informed consent and enrolled in the study

Randomization method use in this study is stratified permuted block randomization, in which trial participants are stratified according to age and gender. Participants will be randomized into three groups, such as acceptance and commitment therapy (ACT) group, mindfulness-based stress reduction (MBSR) group and control group by a research assistant who is not involved in conduct of the study and data analysis in a 1:1:1 allocation ratio. Participants are blinded of the study hypotheses while researchers are blinded of the randomization process and assessment

Control group on waiting list (n= 40)

Mindfulness-based stress reduction (MBSR) group (n= 40)

Acceptance and commitment therapy (ACT) group (n= 40)

Pre-intervention assessment (T1):

All participants were administered with socio-demographic and clinical questionnaire, Posttraumatic Growth Inventory-Short Form (PTGI-SF), Dispositional Hope Scale, Life Orientation Test-Revised (LOT-R), Acceptance and Action Questionnaire (AAQ-II), Hospital Anxiety and Depression Scale (HADS), and Functional Assessment of Cancer Therapy – Head & Neck (FACT-H & N)

No intervention

8 weeks of Mindfulness-based stress reduction (MBSR)

8 weeks of Acceptance and commitment therapy (ACT)

Immediate post-intervention assessment (T2):

All participants were administered with Posttraumatic Growth Inventory-Short Form (PTGI-SF), Dispositional Hope Scale, Life Orientation Test-Revised (LOT-R), Acceptance and Action Questionnaire (AAQ-II), Hospital Anxiety and Depression Scale (HADS), and Functional Assessment of Cancer Therapy – Head & Neck (FACT-H & N)

Reassess after 6 months after intervention (T3):

All participants were administered with Posttraumatic Growth Inventory-Short Form (PTGI-SF), Dispositional Hope Scale, Life Orientation Test-Revised (LOT-R), Acceptance and Action Questionnaire (AAQ-II), Hospital Anxiety and Depression Scale (HADS), and Functional Assessment of Cancer Therapy – Head & Neck (FACT-H & N)

Data analysis

Publication of research findings

**Figure 3. Flowchart of the chronology of research activities in the study**

**Milestones and Dates:**

| Project Milestones (assuming project starts in 1^st^ July 2021) |
| --- |
| **Randomized controlled trial of acceptance and commitment therapy versus mindfulness-based stress reduction in newly diagnosed**  **head and neck cancer patients:**   1. Participant recruitment (n= 120) 2. Randomization of participants into MBSR, ACT and control group   (To be completed in 3 months- up to April 2023) |
| 1. (a) Pre-intervention assessment and data collection   (b) Conducting intervention in MBSR and ACT groups  (c) Immediate post-intervention assessment and data collection  (d) Reassessment and data collection 6 months after intervention  (To be completed in 27 months- up to November 2023) |
| 1. Data analysis:   (a) To examine the changes in the degree of posttraumatic growth (PTG), hope, optimism, quality of life, depression, anxiety, and experiential avoidance of head and neck cancer patients in the acceptance and commitment therapy (ACT) group compared with those in the control group at post-intervention and 6 months after intervention compared with pre-intervention.  (b) To examine the changes in the degree of posttraumatic growth (PTG), hope, optimism, quality of life, depression, anxiety, and experiential avoidance of head and neck cancer patients in the mindfulness-based stress reduction (MBSR) group compared with those in the control group at post-intervention and 6 months after intervention compared with pre-intervention.  (c) To determine whether there are any difference in the changes in the degree of posttraumatic growth (PTG), hope, optimism, quality of life, depression, anxiety, and experiential avoidance between those in the acceptance and commitment therapy (ACT) group and mindfulness-based stress reduction (MBSR) group at post-intervention and 6 months after intervention.  (To be completed in 1 month- up to December 2023) |
| 1. Publication of research findings   (To be completed in 6 months- up to June 2024) |

**Gantt chart:**

| GANTT CHART | ***2021*** | | | | | | ***2022*** | | | | | | | | | | | | | ***2023*** | | | | | | | | | | | | ***2024*** | | | | | |
| --- | --- | --- | --- | --- | --- | --- | --- | --- | --- | --- | --- | --- | --- | --- | --- | --- | --- | --- | --- | --- | --- | --- | --- | --- | --- | --- | --- | --- | --- | --- | --- | --- | --- | --- | --- | --- | --- |
|  | ***J*** | ***A*** | ***S*** | ***O*** | ***N*** | ***D*** | | ***J*** | ***F*** | ***M*** | ***A*** | ***M*** | ***J*** | ***J*** | ***A*** | ***S*** | ***O*** | ***N*** | ***D*** | ***J*** | ***F*** | ***M*** | ***A*** | ***M*** | ***J*** | ***J*** | ***A*** | ***S*** | ***O*** | ***N*** | ***D*** | ***J*** | ***F*** | ***M*** | ***A*** | ***M*** | ***J*** |
| **Randomized controlled trial of acceptance and commitment therapy versus mindfulness-based stress reduction in newly diagnosed head and neck cancer patients (Phase II):**   1. Participant recruitment (n= 120) 2. Randomization of participants into MBSR, ACT and control group |  |  |  |  |  |  | |  |  |  |  |  |  |  |  |  |  |  |  |  |  |  |  |  |  |  |  |  |  |  |  |  |  |  |  |  |  |
| 1. (a) Pre-intervention assessment and data collection   (b) Conducting intervention in MBSR and ACT groups  (c) Immediate post-intervention assessment and data collection  (d) Reassessment and data collection 6 months after intervention |  |  |  |  |  |  | |  |  |  |  |  |  |  |  |  |  |  |  |  |  |  |  |  |  |  |  |  |  |  |  |  |  |  |  |  |  |
| 1. Data analysis:   (a) To examine the changes in the degree of posttraumatic growth (PTG), hope, optimism, quality of life, depression, anxiety, and experiential avoidance of head and neck cancer patients in the acceptance and commitment therapy (ACT) group compared with those in the control group at immediately post-intervention (8 weeks) and 6 months after intervention compared with pre-intervention.  (b) To examine the changes in the degree of posttraumatic growth (PTG), hope, optimism, quality of life, depression, anxiety, and experiential avoidance of head and neck cancer patients in the mindfulness-based stress reduction (MBSR) group compared with those in the control group at immediately post-intervention (8 weeks) and 6 months after intervention compared with pre-intervention.  (c) To determine whether there are any difference in the changes in the degree of posttraumatic growth (PTG), hope, optimism, quality of life, depression, anxiety, and experiential avoidance between those in the acceptance and commitment therapy (ACT) group and mindfulness-based stress reduction (MBSR) group at immediately post-intervention (8 weeks) and 6 months after intervention compared with pre-intervention. |  |  |  |  |  |  | |  |  |  |  |  |  |  |  |  |  |  |  |  |  |  |  |  |  |  |  |  |  |  |  |  |  |  |  |  |  |
| 1. Publication of research findings |  |  |  |  |  |  | |  |  |  |  |  |  |  |  |  |  |  |  |  |  |  |  |  |  |  |  |  |  |  |  |  |  |  |  |  |  |

**Novel theories/New findings/Knowledge:**

To date, the effect of ACT on the PTG, hope, optimism, experiential avoidance, internalized stigma, depression, anxiety, and QoL of head and neck cancer patients have not been investigated. As head and neck cancer patients may experience physical disfigurement and a number of physical complications disrupting their mental well-being leading to poor outcomes of illness, evidence indicating the efficacy of MBSR and ACT to enhance positive psychology and QoL while diminishing internalized stigma revealed the importance of mindfulness-based intervention to safeguard the mental well-being of head and neck cancer patients. If MBSR and ACT are effective to facilitate mental well-being of head and neck cancer patients, then these psychosocial interventions should be included as part of the treatment regime for this group of cancer patients.

**Specific or Potential Applications:**

(1) If proven efficacious, ACT can be utilized as an individual or group psychosocial intervention while MBSR as group psychosocial intervention which not only decrease psychological complications but also increase positive psychology and QoL of head and neck cancer patients. Hence, this justified the inclusion of ACT and MBSR as part of the treatment regime for head and neck cancer patients.

(2) The methodology of this study can be replicated to investigate the effect of ACT and MBSR on psychological complications and other positive psychology in head and neck cancer patients and other types of cancer.

**Ethical Issues:**

(a) Ethical approval will be obtained from the human ethical committee of USM.

(b) The research team respect the wishes of subjects that do not wish to be enrolled in the study. Subjects who do not want to participate can request to “opt-out” and we will not use any of the information collected.

(c) Issues of confidentiality and privacy measures:

Subjects will be explained that the result will only be used for the purpose of research and will not be recorded in the patient’s BHT.

(d) Subjects are allowed to withdraw at any time during the study without specifying the reason and we will not use any of the information collected.

(e) There is no conflict of interest for the research team.

(f) Subjects’ personal identifiable information will not be elicited and they are assured of their participation anonymity. Each subject will be given research number eg RCT001, etc. All the documents involved in assessment of all subjects including subject’s personal information (socio-demographic, substance history, symptomatology, and response to questionnaires) are kept in document files and locked in a cabinet with the key kept by the primary investigator. Only the primary investigator and co-researchers are allowed to access the files for data analysis and for publication purposes. The files will be kept for duration of 7 years after completion of the study and then it will be destroyed completely.

(g) As a token of appreciation, respondents will be compensated with honorarium of RM 20 for each assessment for their willingness and time spent on the study (each assessment session is approximately 20 minutes). Therefore, duration of each subject participation in the study is only 20 minutes in each assessment and total duration of participation is 1 hour.

(h) Risk of the study to the subjects:

Some of the questions asked in the study may cause some subjects to be disturbed. All subjects developed any mental disturbances while participating in the study, they will be recommended for referrals to Department of Psychiatry, Hospital Bukit Mertajam, Hospital Pulau Pinang and Universiti Kebangsaan Malaysia Medical Centre (nearest psychiatric service available). In addition, prior to referral, rescue medication such as benzodiazepine will be administered to calm down patients.

(i) The subjects may benefit from the study as we provide information regarding the mental health of the subjects and any subjects found to have depression and anxiety disorders will also be recommended for referrals to Klinik Minda Sihat, AMDI, USM, Department of Psychiatry, Hospital Bukit Mertajam, Hospital Pulau Pinang and Universiti Kebangsaan Malaysia Medical Centre for further treatment and follow up. The community will also benefit from the study findings as we provide scientific evidence regarding the effects of two psychosocial interventions i.e. ACT and MBSR on the positive psychology and stigma of head and neck cancer patients.

(j) Adverse event report:

Subjects may also withdraw from the study should an adverse event occurs. Adverse event (AE) is any untoward medical occurrence in a subject administered a trial intervention that does not necessarily have a causal relationship with this treatment. An AE can be any unfavorable and unintended sign, symptom, or disease temporarily associated with the use of investigational intervention, whether or not related to the investigational intervention. Subjects will be issued with a study card with the contact details of the research team and they are encouraged to maintain close contact by phone, to report any AE occurring in subjects. If the case of adverse event (AE), the event is reported in the adverse event section in the Case report form (CRF) and serious adverse event report is filled if necessary (Figure 5). The details to be reported include the name of the event, date of onset and date of recovery, severity, relationship to the study treatment, measures taken regarding study treatment, treatment of adverse event and outcome of the event (resolved/ongoing). Some reasons that may lead to subjects withdrawing from the study in cases of AE are:

- Presence of adverse reactions not related to the study, but subjects feel uncomfortable to continue this study.
- Presence of adverse reactions that may be related to the study, such as unusual illnesses which started upon this intervention.
- Unusual changes in behavior, temperament, routine, etc of subjects, which started upon this intervention.
- Any suspected or unexpected adverse events that is not consistent with the general acceptance of the ACT and MBSR administration. In general, psychotherapy should not exhibit any side effects or health detrimental effects.

**Occurrence of an adverse event (Unexpected or expected)**

**Report to investigators**

**Documentation by investigators:**

- Fill in Case Report Form (CRF)
- If necessary, fill in Serious Adverse Event Report (death, hospitalization)

**Case assessment**

**Non-SUSAR**

**Suspected Unexpected Serious Adverse Report (SUSAR)**

**Report to ethics committee**

**Clinical Trial Database**

**Clinical Study Report**

**Figure 4. Flow chart of adverse event**

**Operational definitions:**

(1) Experiential avoidance: Experiential avoidance (EA) has been broadly defined as attempts to avoid thoughts, feelings, memories, physical sensations, and other internal experiences—even when doing so creates harm in the long-run.

(2) Positive psychology: Positive psychology is defined as a scientific approach to study human thoughts, feelings and behaviours which focus on the strength and the good outcomes it brought in life which allow others to progress in life instead of just resulting in one who is struggling in life to improve his/her life up to where it was before the struggle.

(3) Posttraumatic growth: Posttraumatic growth (PTG) is positive psychological changes experienced by a person as a result of struggle due to life-threatening crisis or event.

(4) Hope: Hope is a positive goal-directed motivational state and it is also a dispositional trait which enables one to have a tendency to adopt a positive outlook in life.

(5) Optimism: Optimism is the stable and consistent believe that good things rather than bad things will happen in one’s life.

(6) Quality of life: Quality of life is the perception of one’s position in life in the context of the culture and values of the community one lives in and in relation to standard, goals, concern and expectations.

(7) Acceptance and commitment therapy: It is a third generation cognitive behavioral approach which uses acceptance and mindfulness processes, and commitment and behavior change processes to produce psychological flexibility. The latter is defined as the ability to contact the present moment more fully as a conscious human being and to change or persist in behavior when doing so serves valued ends.

(8) Mindfulness-based stress reduction: The term ‘mindfulness’ refers to mindful awareness as a way of being – a knowing and experiencing of feelings, thoughts, and perceptions as they arise and pass away each moment. It is a way of relating to all experiences in an open, receptive way, without judging experiences as good or bad (grasping at them or pushing them away). Mindfulness-based stress reduction is an 8-week, standardized group intervention consisting of mindfulness meditation and gentle yoga that is designed to have applications for stress, pain, and illness.

**Registration:**

This clinical trial is registered with US National Library of Medicine ClinicalTrials.gov with a ClinicalTrials.gov identifier (NCT number): NCT04800419 (<https://clinicaltrials.gov/ct2/show/NCT04800419>).

**References:**

Aguirre-Camacho, A., Pelletier, G., González-Márquez, A., The relevance of experiential avoidance in breast cancer distress: insights from a psychological group intervention. Psychooncology. 26(4), 469-475.

Birnie, K., Speca, M., Carlson, L. E. (2010). Exploring self-compassion and empathy in the context of mindfulness-based stress reduction (MBSR). Stress and Health. 26, 359–371.

Bishop, S. R., Lau, M., Shapiro, S., et al. (2004). Mindfulness: A proposed operational definition. Clinical Psychology: Science and Practice. 11, 230–241.

Bjelland, I., Dahl, A. A, Haug, T. T., et al. (2002). The validity of the Hospital Anxiety and Depression Scale. An updated literature review. Journal of Psychosomatic Research. 52(2), 69-77.

Bond, F. W., Hayes, S. C., Baer, R. A., et al. (2011). Preliminary psychometric properties of the Acceptance and Action Questionnaire-II: a revised measure of psychological inflexibility and experiential avoidance. Behavior Therapy. 42(4), 676-688.

Bränström, R., Kvillemo, P., Brandberg, Y., et al. (2010). Self-report mindfulness as a mediator of psychological well-being in a stress reduction intervention for cancer patients – A randomized study. Annals of Behavioral Medicine. 39, 151–161.

Bryant, F. B., Yarnold, P. R. (1995). Principal-components analysis and exploratory and confirmatory factor analysis. In L. G. Grimm & P. R. Yarnold (Eds.), Reading and understanding multivariate statistics (pp. 99-136). American Psychological Association: Washington, DC.

Brown Johnson, C. G., Brodsky, J. L., Cataldo, J. K. (2014). Lung cancer stigma, anxiety, depression, and quality of life. Journal of Psychosocial Oncology. 32(1), 59–73.

Cann, A., Calhoun, L. G., Tedeschi, R. G. (2010). A short form of Posttraumatic Growth Inventory. Anxiety, Stress and Coping. 23(2), 127-137.

Carmody, J., Baer, R. A. (2008). Relationships between mindfulness practice and levels of mindfulness, medical and psychological symptoms and well-being in a mindfulness-based stress reduction program. Journal of Behavioral Medicine. 31, 23–33.

Casellas‐Grau, A., Ochoa, C. Ruini, C. (2017). Psychological and clinical correlates of posttraumatic growth in cancer: A systematic and critical review. Psycho‐oncology. 26(12), 2007-2018.

Cataldo, J. K., Brodsky, J. L. (2013). Lung cancer stigma, anxiety, depression and symptom severity. Oncology. 85(1), 33-40.

Cella, D. F., Tulsky, D. S., Gray, G., et al. (1993). The Functional Assessment of Cancer Therapy scale: development and validation of the general measure. Journal of Clinical Oncology. 11(3), 570-579.

Chang, L. C., Li, I. C., (2002). The correlation between perceptions of control and hope status in home-based cancer patients. The Journal of Nursing Research. 10(1), 73-82.

Cho, J., Choi, E. K., Kim, S. Y., et al. (2013). Association between cancer stigma and depression among cancer survivors: a nationwide survey in Korea. Psycho-oncology. 22(10), 2372-2378.

Cho, D., Park, C. L. (2013). Growth following trauma: Overview and current status. Terapia psicológica. 31(1), 69–79.

Committee on Developing Evidence-Based Standards for Psychosocial Interventions for Mental Disorders; Board on Health Sciences Policy; Institute of Medicine; et al. (2015). Psychosocial Interventions for Mental and Substance Use Disorders: A Framework for Establishing Evidence-Based Standards. Washington (DC): National Academies Press (US). <https://www.ncbi.nlm.nih.gov/books/NBK321284/> (Accessed 21 January 2021).

Corrigan, P. W., Kerr, A., Knudsen, L. (2005). The stigma of mental illness: Explanatory models and methods for change. Applied and Preventive Psychology. 11, 179-190.

Donovan-Kicken, E., Caughlin, J. P. (2011). Breast cancer patients’ topic avoidance and psychological distress: The mediating role of coping. Journal of Health Psychology. 16(4), 596-606.

Doss, J. G., Thomson, W. M., Drummond, B. K., et al. (2011). Validity of the FACT-H&N (v 4.0) among Malaysian oral cancer patients. Oral Oncology. 47(2011), 648–652.

Dropkin, M. J. (1999). Body image and quality of life after head and neck cancer surgery. Cancer Practice. 7, 309–313.

Eisenberg, D., Downs, M. F., Golberstein, E., et al. (2009). Stigma and help seeking for mental health among college students. Medical Care Research and Review. 66, 522-541.

Ernst, J., Mehnert, A., Dietz, A. et al. (2017). Perceived stigmatization and its impact on quality of life - results from a large register-based study including breast, colon, prostate and lung cancer patients. BMC Cancer. 17, 741.

Everson, S. A., Goldberg, D. E., Kaplan, G. A., et al. (1996). Hopelessness and risk of mortality and incidence of myocardial infarction and cancer. Psychosomatic Medicine. 58, 113-121.

Feelemyer, J. P., Jarlais, D. C. D., Arasteh, K., et al. (2014). Changes in quality of life (WHOQOLBREF) and addiction severity index (ASI) among participants in opioid substitution treatment (OST) in low and middle income countries: An international systematic review. Drug and Alcohol Dependence. 134, 251-258.

Feros, D. L., Lane, L., Ciarrochi, J., et al. (2013). Acceptance and commitment therapy (ACT) for improving the lives of cancer patients: A preliminary study. Psycho-Oncology. 22(2), 459-464.

Garland, S. N., Carlson, L. E., Cook, S., et al. (2007). A non-randomized comparison of mindfulnessbased stress reduction and healing arts programs for facilitating post-traumatic growth and spirituality in cancer outpatients. Supportive Care in Cancer. 15, 949–961.

Hagedoorn, M., Molleman, E. (2006). Facial disfigurement in patients with head and neck cancer: the role of social self-efficacy. Health Psychology. 25(5), 643-647.

Han, Y., Yuan, J., Luo, Z., et al. (2013). Determinants of hopelessness and depression among Chinese hospitalized esophageal cancer patients and their family caregivers. Psycho‐Oncology. 22(11), 2529-2536.

Hawkes, A. L., Pakenham, K. I., Chambers, S. K., et al. (2014). Effects of a multiple health behavior change intervention for colorectal cancer survivors on psychosocial outcomes and quality of life: a randomized controlled trial. Annals of Behavioral Medicine. 48, 359–370.

Hayes, S. C., Wilson, K. G., Gifford, E. V., et al. (1996). Experiential avoidance and behavioral disorders: A functional dimensional approach to diagnosis and treatment. Journal of Consulting and Clinical Psychology. 64(6), 1152–1168.

Hayes, S. C., Strosahl, K. D., Wilson, K. G. (1999). Acceptance and commitment therapy: An experiential approach to behaviour change. The Guilford Press: New York.

Hayes, S. C., Luoma, J. B., Bond, F. W., et al. (2006). Acceptance and commitment therapy: model, processes and outcomes. Behaviour Research and Therapy. 44(1), 1–25.

Henry, J. L., Wilson, P. H., Bruce, D. G., et al. (1997). Cognitive-behavioural stress management for patients with noninsulin dependent diabetes mellitus. Psychology, Health & Medicine. 2(2), 109-118.

Ho, S., Rajandram, R. K., Chan, N., et al. (2011). The roles of hope and optimism on the posttraumatic growth in oral cavity cancer patients. Oral Oncology. 47(2011), 121-124.

Hou, W. K., Law, C. C., Yin, J., et al. (2010). Resource loss, resource gain, and psychological resilience and dysfunction following cancer diagnosis: a growth mixture modeling approach. Health Psychology. 29(5), 484-495.

Holtmaat, K., van der Spek, N., Cuijpers, P., et al. (2017). Posttraumatic growth among head and neck cancer survivors with psychological distress. Psycho-Oncology. 26, 96-101.

Horney, D. J., Smith, H. E., McGurk, M., et al. (2011). Associations between quality of life, coping styles, optimism, and anxiety and depression in pretreatment patients with head and neck cancer. Head & neck. 33(1), 65-71.

Humphris, G. M., Rogers, S., McNally, D., et al. (2003). Fear of recurrence and possible cases of anxiety and depression in orofacial cancer patients. International Journal of Oral Maxillofacial Surgery. 32, 486–491.

Ibrahim, N. M., Shohaimi, S., Chong, H. T., et al. (2009). Validation study of the Mini-Mental State Examination in a Malay-speaking elderly population in Malaysia. Dementia Geriatric Cognitive Disorder. 27(3), 247-253.

Jo, K. H., Son, B. K. (2004). The relationship of uncertainty, hope and quality of life in patients with breast cancer. Journal of Korean Academy of Nursing. 34(7), 1184-1193.

Johns, S. A., Stutz, P. V., Talib, T. L., et al. (2019). Acceptance and commitment therapy for breast cancer survivors with fear of cancer recurrence: a 3-arm pilot randomized controlled trial. Cancer. 126, 211-218.

Kabat-Zinn, J. (1990). Full catastrophe living: Using the wisdom of your body and mind to face stress, pain and illness. Delacourt : New York, NY.

Karakoyun-Celik, O., Gorken, I., Sahin, S., et al. (2010). Depression and anxiety levels in woman under follow-up for breast cancer: relationship to coping with cancer and quality of life. Medical Oncology. 27, 108-113.

Kim, S.W., Kim, S.Y., Kim, J.M., et al. (2011). Relationship between a hopeful attitude and cellular immunity in patients with breast cancer. General Hospital Psychiatry. 33(4), 371-376.

King, S., Exley, J., Parks, S., et al. (2016). The use and impact of quality of life assessment tools in clinical care settings for cancer patients, with a particular emphasis on brain cancer: insights from a systematic review and stakeholder consultations. Quality of Life Research. 25(9), 2245–2256.

Kugaya, A., Akechi, T., Okuyama, T., et al. (2000). Prevalence, predictive factors and screening for psychological distress in patients with newly diagnosed head and neck cancer. Cancer. 88, 2817-2823.

Kvillemo, P., Bränström, R. (2010). Experiences of a mindfulness-based stress-reduction intervention among patients with cancer. Cancer Nursing. 34, 24–31.

Labelle, L. E., Lawlor-Savage, L., Campbell, T. S., et al. (2015). Does self-report mindfulness mediate the effect of Mindfulness-based stress reduction (MBSR) on spirituality and posttraumatic growth in cancer patients? The Journal of Positive Psychology. 10(2), 153-166.

Lebel, S., Castonguay, M., Mackness, G., et al. (2011). The psychosocial impact of stigma in people with head and neck or lung cancer. Psycho-oncology. 22(1), 140-152.

Lecrubier, Y., Sheehan, D. V., Weiller, E., et al. (1997). The Mini International Neuropsychiatric Interview (MINI). A short diagnostic structured interview: reliability and validity according to the CIDI. European Psychiatry. 12(5), 224-231.

Lengacher, C. A., Johnson-Mallard, V., Post-White, J., et al. (2009). Randomized controlled trial of mindfulness-based stress reduction (MBSR) for survivors of breast cancer. PsychoOncology. 18, 1261–1272.

Leong Abdullah, M. F. I., Nik Jaafar, N. R., Zakaria, H., et al. (2015). Posttraumatic growth, depression and anxiety in head and neck cancer patients: examining their patterns and correlations in a prospective study. Psycho-Oncology. 24(8), 894-900.

Leong Abdullah, M. F. I., Hami, R., Appalanaido, G. K., et al. (2017a). Validation of the Malay version of the Posttraumatic Growth Inventory-Short Form (PTGI-SF) among Malaysian cancer patients. ASEAN Journal of Psychiatry. 18(2), 135-143.

Leong Bin Abdullah, M. F. I., Hami, R., Appalanaido, G. K., et al. (2017b). Validation of the Malay version of the Life Orientation Test-Revised (LOT-R) among Malaysian cancer patients. Journal of Biomedical and Clinical Sciences. 2(2), 8-13.

Leong Bin Abdullah, M. F. I., Hami, R., Appalanaido, G. K., et al. (2018). Validation of the Malay version of the Hope Scale among Malaysian cancer patients. Malaysian Journal of Health Sciences. 16(1), 95-102.

Leong Abdullah, M.F.I., Hami, R., Appalanaido, G.K., et al. (2019). Diagnosis of cancer is not a death sentence: Examining posttraumatic growth and its associated factors in cancer patients. Journal of Psychosocial Oncology. 37(5), 636-651.

Lin, C. C., Tsay, H. F. (2005). Relationships among perceived diagnostic disclosure, health locus of control, and levels of hope in Taiwanese cancer patients. Psycho‐Oncology: Journal of the Psychological. Social and Behavioral Dimensions of Cancer. 14(5), 376-385.

Long, S. A., D’Antonio, L. L., Robinson, E. B., et al. (1996). Factors related to quality of life and functional status in 50 head and neck cancer patients. Laryngoscopy. 106, 1084-1088.

Lua, P. L., Wong, S.Y. (2012). The reliability of the Malay versions of Hospital Anxiety and Depression Scale (HADS) and Mcgill Quality of Life Questionnaire (MQOL) among a group of patients with cancer in Malaysia. Malaysian Journal of Psychiatry Online. 21(1), 1-13. http://www.mjpsychiatry.org/index.php/mjp/article/viewFile/178/138 (accessed 2 March 2021).

Mackenzie, M. J., Carlson, L. E., Munoz, M., et al. (2007). A qualitative study of self-perceived effects of mindfulness-based stress reduction (MBSR) in a psychosocial oncology setting. Stress and Health. 23, 59–69.

Massie, M. J. (2004). Prevalence of depression in patients with cancer. Journal of the National Cancer Institute Monograph. 32, 57–71.

Michaels, P. J., López, M., Rüsch, N. (2017). Constructs and concepts comprising the stigma of mental illness. Psychology, Society, & Education. 4, 183-194.

Miller, D. L., Manne, S. L., Taylor, K., et al. (1996). Psychological distress and well-being in advanced cancer: The effects of optimism and coping. Journal of Clinical Psychology in Medical Settings. 3(2), 115-130.

Mukhtar, F., Abu Bakar, A. K., Mat Junus, M., et al. (2012). A preliminary study on the specificity and Sensitivity values and inter-rater reliability of Mini international neuropsychiatric Interview (MINI) in Malaysia. ASEAN Journal of Psychiatry. 13(2).

Ng, X., Lau, Y., Klainin-Yobas, P. (2020). The effectiveness of mindfulness-based interventions among cancer patients and survivors: a systematic review and meta-analysis. Supportive Care in Cancer. 28, 1563–1578.

Páez, M., Luciano, M. C., Gutiérrez, O. (2007). Psychological treatment for breast cancer. Comparison between acceptance based and cognitive control based strategies. Psicooncología. 4, 75-95.

Pangman, V. C., Sloan, J., Guse, L. (2000). An examination of Psychometric Properties of the Mini-Mental State Examination and the Standardised Mini-Mental State Examination: Implications for Clinical Practice. Applied Nursing Research. 13(4), 209-213.

Pescosolido, B. A., Martin, J. K. (2015). The stigma complex. Annual review of sociology. 41, 87-116.

Petersen, L. R., Clark, M. M., Novotny, P., et al. (2008). Relationship of optimism–pessimism and health-related quality of life in breast cancer survivors. Journal of Psychosocial Oncology. 26(4), 15-32.

Pollard, A., Burchell, J. L., Castle, D., et al. (2017). Individualised mindfulness‐based stress reduction for head and neck cancer patients undergoing radiotherapy of curative intent: a descriptive pilot study. Cancer Care. 26(2), e12474.

Raingruber, B. (2011). The effectiveness of psychosocial interventions with cancer patients: an integrative review of the literature (2006–2011). International Scholarly Research Notices. 2011, 638218.

Ramli, M., Mohd Ariff, F., Khalid, Y., et al. (2008). Validation of the Bahasa Malaysia version of the Coping Inventory for Stressful Situation. Malaysian Journal of Psychiatry Online. 17(2), 1-8.

Ryu, Y. M., Yi, M. (2013). The Factors Influencing Quality of Life in Women with Breast Cancer. Asian Oncology Nursing. 13(3), 121-127.

Scheier, M. F., Carver, C. S., Bridges, M. W. (1994). Distinguishing optimism from neuroticism (and trait anxiety, self-mastery, and self-esteem): A re-evaluation of the Life Orientation Test. Journal of Personality and Social Psychology. 67, 1063-1078.

Shand, L.K., Cowlishaw, S., Brooker, J.E., et al. (2015). Correlates of post‐traumatic stress symptoms and growth in cancer patients: A systematic review and meta‐analysis. Psycho‐Oncology. 24(6), 624-634.

Sharp, L., Redfearn, D., Timmons, A., et al. (2018). Posttraumatic growth in head and neck cancer survivors: is it possible and what are the correlates? Psycho-Oncology. 27(6):1517-1523.

Shapiro, S. L., Carlson, L. E. (2009). The art and science of mindfulness: Integrating mindfulness into psychology and the helping professions. American Psychological Association: Washington, DC.

Shari, N. I., Zainal, N. Z., Guan, N C., et al. (2019). Psychometric properties of the acceptance and action questionnaire (AAQ II) Malay version in cancer patients. PLoS ONE. 14(2), e0212788.

Sheehan, D. V., Lecrubier, Y., Sheehan, K. H., et al. (1998). The Mini-International Neuropsychiatric Interview (M.I.N.I.): the development and validation of a structured diagnostic psychiatric interview for DSM-IV and ICD-10. Journal of Clinical Psychiatry. 59(20:22-33), 34-35.

Shimizu, K., Nakaya, N., Saito-Nakaya, et al. (2012). Clinical biopsychosocial risk factors for depression in lung cancer patients: a comprehensive analysis using data from the Lung Cancer Database Project. Annals of Oncology. 23(8), 1973-1979.

Smith, E. M., Gomm, S. A., Dickens, C. M. (2003). Assessing the independent contribution to quality of life from anxiety and depression in patients with advanced cancer. Palliative Medicine. 17, 509-513.

Snyder, C.R., Harris, C., Anderson, et al. (1991). The will and the ways: development and validation of an individual-differences measure of hope. Journal of Personality and Social Psychology. 60(4), 570-585.

Spiegel, D., Giese-Davis, J. (2003). Depression and cancer: mechanism and disease progression. Biological Psychiatry. 54, 269-282.

Strauss, R. P. (1989). Psychosocial responses to oral and maxillofacial surgery for head and neck cancer. Journal of Oral Maxillofacial Surgery. 47, 343–348.

Stuber, J., Meyer, I., Link, B. (2008). Stigma, prejudice, discrimination and health. Social Science & Medicine. 67, 351-357.

Tedeschi, R. G., Calhoun, L. G. (1996). The Posttraumatic Growth Inventory: measuring the positive legacy of trauma. Journal of Trauma and Stress. 9(3), 455-471.

Tedeschi, R. G., Calhoun, L. G. (2004). Posttraumatic growth: Conceptual foundations and empirical evidence. Psychological inquiry. 15(1), 1-18.

The Star. (2017). Research on head and neck cancers promising. <https://www.thestar.com.my/opinion/letters/2017/06/22/research-on-head-and-neck-cancers-promising/> (Accessed 21 January 2021).

Threader, J., McCormack, L. (2016). Cancer-related trauma, stigma and growth: the 'lived' experience of head and neck cancer. European Journal of Cancer Care. 25(1), 157-169.

Tomich, P.L., Helgeson, V.S. (2012). Posttraumatic growth following cancer: Links to quality of life. Journal of Traumatic Stress. 25(5), 567-573.

Tuijl, J. P., Scholte, E. M., de Craen, A. J. M. (2012). Screening for cognitive impairment in older General hospital patients: comparison of the six-item cognitive test with the Mini-Mental State Examination. International Journal of Geriatric Psychiatry. 27, 755-762.

World Health Organization (2014). Locally advanced squamous carcinoma of the head and neck. <https://www.who.int/selection_medicines/committees/expert/20/applications/HeadNeck.pdf> (Accessed 20 January 2021).

Yang, Y.L., Liu, L., Wang, X.X., et al. (2014). Prevalence and associated positive psychological variables of depression and anxiety among Chinese cervical cancer patients: a cross-sectional study. PloS one. 9(4), p.e94804.

Yong, H. W., Hashim, Z., Jamil @ Osman, Z. (2016). Reliability and validatidity of Hospital Anxiety and Depression Scale (HADS) on breast cancer survivors: Malaysia case study. Asia Pacific Environmental and Occupational Health Journal. 2(2), 19-24.

Yanos, P. T., Roe, D., Markus, K., et al. (2008). Pathways between internalized stigma and outcomes related to recovery in schizophrenia spectrum disorders. Psychiatric Services. 59, 1437-1442.

Yi, J., Zebrack, B., Kim, M. A., et al. (2015). Posttraumatic growth outcomes and their correlates among young adult survivors of childhood cancer. Journal of Pediatric Psychology. 40(9), 981-991.

Yusoff, N., Low, W. Y., Yip, C. H. (2010). The Malay Version of the European Organization for Research and Treatment of Cancer Quality of Life Questionnaire (EORTC-QLQ C30): Reliability and Validity Study. The International Medical Journal Malaysia. 9(2), 45-50.

Zhao, C., Lai, L., Zhang, L., et al. (2021). The effects of acceptance and commitment therapy on the psychological and physical outcomes among cancer patients: A meta-analysis with trial sequential analysis. Journal of Psychosomatic Research. 140, 110304.

Zigmond, A. S., Snaith, R. P. (1983). The hospital anxiety and depression scale. Acta Psychiatrica Scandinavica. 67(6), 361-370.

**Budget:**

| **Vote**  *Vot* | **Budget Details & Justification**  *Perincian Bajet & Justifikasi* | **Amount requested by researcher (RM)**  *Amaun yang dipohon oleh penyelidik (RM)* | | |
| --- | --- | --- | --- | --- |
|  |  | **Year 1**  *Tahun 1* | **Year 2**  *Tahun 2* | **Year 3**  *Tahun 3* |
| **11000**  Salary and wages  *Gaji dan upah* |  |  |  |  |
| **21000**  Travelling expenses  and subsistence  *Perbelanjaan Perjalanan dan Sara Hidup* | Travelling, accommodation and meal expenses for setting up and data discussion with UKMMC:  (1) Penang-KL-Penang airfare (RM300 x 1 person x 3 trips)  (2) Accommodation (RM220 x 3 nights x 1 person x 3 trips)  (3) Food allowance (RM85/day x 4 days x 1 person x 3 trips)  Travelling, accommodation and meal expenses for local conference:  (1) Penang-KL-Penang airfare (RM300 x 1 person)  (2) Accommodation (RM220 x 3 nights x 1 person)  (3) Food allowance (RM85/day x 4 days x 1 person) | 600  1320  680 | 300  660  340 | 300  660  340 |
| **23000**  Communication and Utilities (Phone,Fax, Postage etc)  *Perhubungan dan Utiliti*  *(Tel,Faks,Pos, dll)* | - | - | - | - |
| **24000**  Rental  *Sewaan* |  | - | - | - |
| **27000**  Research Materials & Supplies (including Animals, Plants, Disposables, etc.)  *Bekalan & Bahan-bahan Lain (termasuk Haiwan, Pokok dan Bahan Pakai Habis)* | Stationary:  • A4 Paper (5 rims) @ RM12 per rim: RM12 x 5 rims= RM 60  • Files for subjects:  RM3 x 247= RM741 | 36  741 | 24  - | - |
| **28000**  Maintenance and Minor Repair Services  *Penyelenggaraan dan Pembaikan Kecil* | - | - | - | - |
| **29000**  Professional services & other services (printing & hospitality, honorarium, bank charges, software license renewals, conference fee, journals page charges, proof reading)  *Perkhidmatan ikhtisas dan perkhidmatan lain-lain (percetakan hospitaliti, honorarium, caj bank, pembaharuan lesen perisian, yuran persidangan & penerbitan, pembacaan pruf)* | (1) Printing:  (a) Photostat copy of questionnaires for Phase I study: at RM0.10 per page x 30 pages x 157 subjects for 1 assessment=  RM 471  (b) Photostat copy of questionnaires for Phase II study: at RM0.10 per page x 30 pages x 120 subjects = RM 360 per assessment x 3 assessments  (2) Honorarium: RM 20 x 3 assessments x 120 subjects= RM 7200  Local conference fees:  Free paper presentation in Malaysian Conference in Psychological Medicine in Kuala Lumpur:  RM1,400 x 1 person | 471  720  RM 3600 | 360  RM 3600 | 1400 |
| **35000 (> RM1,000)**  Equipment[Please provide 3 quotations]  *Peralatan [Sila berikan 3 sebut harga]* | - | - | - | - |
| **52000**  Other payment such as bank charges, software license renewals and taxes.  *Bayaran-bayaran lain seperti pembaharuan lesen (cth: lesen pengangkutan) dan cukai.* | - | - | - | - |
| **TOTAL AMOUNT** | | **8,168** | **5,284** | **2,700** |
| ***GRAND AMOUNT** | | **16,152** | | |
